# Eradicating mesothelin-positive human gastric and pancreatic tumors in xenograft models with optimized anti-mesothelin antibody–drug conjugates from synthetic antibody libraries

**DOI:** 10.1038/s41598-021-94902-1

**Published:** 2021-07-29

**Authors:** Hung-Ju Hsu, Chao-Ping Tung, Chung-Ming Yu, Chi-Yung Chen, Hong-Sen Chen, Yu-Chuan Huang, Pei-Hsun Tsai, Su-I Lin, Hung-Pin Peng, Yi-Kai Chiu, Yueh-Liang Tsou, Wei-Ying Kuo, Jhih-Wei Jian, Fei-Hung Hung, Chiao-Yun Hsieh, Michael Hsiao, Simon Shih-Hsien Chuang, Chia-Ning Shen, Yong Alison Wang, An-Suei Yang

**Affiliations:** 1 Genomics Research Center, Academia Sinica, 128 Academia Rd., Sec. 2, Nankang Dist., Taipei, 115 Taiwan; 2 Institute of Pharmaceutics, Development Center for Biotechnology, Taipei, 115 Taiwan; 3 Koo Foundation Sun Yat-Sen Cancer Center, Taipei, 112 Taiwan

**Keywords:** Drug development, Molecular engineering

## Abstract

Mesothelin (MSLN) is an attractive candidate of targeted therapy for several cancers, and hence there are increasing needs to develop MSLN-targeting strategies for cancer therapeutics. Antibody–drug conjugates (ADCs) targeting MSLN have been demonstrated to be a viable strategy in treating MSLN-positive cancers. However, developing antibodies as targeting modules in ADCs for toxic payload delivery to the tumor site but not to normal tissues is not a straightforward task with many potential hurdles. In this work, we established a high throughput engineering platform to develop and optimize anti-MSLN ADCs by characterizing more than 300 scFv CDR-variants and more than 50 IgG CDR-variants of a parent anti-MSLN antibody as candidates for ADCs. The results indicate that only a small portion of the complementarity determining region (CDR) residues are indispensable in the MSLN-specific targeting. Also, the enhancement of the hydrophilicity of the rest of the CDR residues could drastically increase the overall solubility of the optimized anti-MSLN antibodies, and thus substantially improve the efficacies of the ADCs in treating human gastric and pancreatic tumor xenograft models in mice. We demonstrated that the in vivo treatments with the optimized ADCs resulted in almost complete eradication of the xenograft tumors at the treatment endpoints, without detectable off-target toxicity because of the ADCs’ high specificity targeting the cell surface tumor-associated MSLN. The technological platform can be applied to optimize the antibody sequences for more effective targeting modules of ADCs, even when the candidate antibodies are not necessarily feasible for the ADC development due to the antibodies’ inferior solubility or affinity/specificity to the target antigen.

## Introduction

Targeting mesothelin (MSLN) is an attractive cancer therapeutic strategy, which has led to a large number of clinical trials of treating diverse cancers^1,2^. MSLN is known to be overexpressed in several malignant tumor cells^3^. At molecular level, the interaction of MSLN with CA-125/MUC16, which participates in cell-to-cell interactions enabling tumorigenesis and tumor proliferation, increases the motility and invasion of pancreatic carcinoma cells^4–7^. The overexpression of MSLN activates NFκB, MAPK, and PI3K pathways, leading to resistance of apoptosis in pancreatic cancer cells^8^. Also, MSLN overexpression results in MMP-7 activation associated with pancreatic carcinoma cell invasion^7^, and MSLN overexpression correlates with higher MMP-9 expression in malignant pleural mesothelioma, promoting tumor invasion^9^. On the one hand, clinical observations indicate that elevated MSLN expression is associated with increase in tumor burden and poor overall survival in patients of various cancers^10–16^, and on the other hand, MSLN’s normal expression is limited to mesothelial cells, which are dispensable without substantial adverse side effects. As such, MSLN is an attractive candidate of targeted therapy for several cancers^2^, and hence there are increasing needs to develop MSLN-targeting strategies for cancer therapeutics.

Antibody–drug conjugates (ADCs) targeting MSLN have been demonstrated to be a viable strategy in treating MSLN-positive cancers^1–3,17–21^. However, developing antibodies as targeting modules in ADCs for toxic payload delivery to the tumor site but not to normal tissues is not a straightforward task, with many potential hurdles. In principle, successful ADC development should meet the following minimal criteria: (1) feasibility of preparing the ADCs with sufficient yield; (2) appropriate affinity and specificity of the ADCs binding to the target antigen; (3) epitope accessibility for ADC binding in a biologically relevant state; (4) ADC internalization in cells following binding to an appropriate epitope; (5) release of toxic payload after the receptor-mediated endocytosis of the ADC-antigen complex^22,23^. In practice, antibody candidates simultaneously satisfying all the criteria above are difficult to attain, and hence optimization of candidate antibodies is essential for successful ADC development.

We have established a high throughput antibody engineering platform for developing antibodies suitable for ADC development. Determining the potency of the antibodies as targeting modules in immunoconjugates has been a low-throughput process due to the rate-limiting step of the antibody-payload conjugation. We accelerated the rate-limiting process of antibody potency determination by high-throughput cytotoxicity screening of non-covalently assembled immunotoxins, which can be easily prepared by mixing an adaptor-toxin fusion protein with secreted soluble synthetic scFvs (single chain variable fragments) in culture medium^24^. This platform has been validated to evaluate the applicability of our synthetic antibody libraries^24–28^ in developing ADCs targeting HER2-overexpressed cancer cells^24,29,30^. The principles governing the efficiency of the antibodies as targeting modules have been elucidated from the cytotoxicity data derived with the high-throughput experimental measurements. We have found that screening against the target cells with a large pool of antibodies from the synthetic antibody libraries without the limitations of natural antibody responses can lead to optimal potency and minimal off-target toxicity of ADCs^24,29,30^.

In this work, we applied the high throughput ADC development platform to develop anti-MSLN ADCs with high potency in eliminating xenograft tumors in mice. We first selected and screened antibody candidates from the synthetic antibody libraries established in our laboratory to attain a candidate anti-MSLN antibody M9^25^. We found that M9 was highly effective in inducing receptor-mediated endocytosis through MSLN binding, but was difficult to conjugate to cytotoxic drugs with high yield because of the low solubility of the antibody drug conjugates. To improve the solubilities of the ADCs, we developed a general procedure to optimize the CDR sequences of the antibody candidates as targeting modules for ADCs, while maintaining the specificity of the M9-derived antibodies to the same epitope on MSLN as that of M9. The analysis of the antibody CDR sequences of the CDR-variants of M9 indicates that only a small portion of the CDR residues are indispensable in the MSLN binding. The enhancement of the hydrophilicity of the rest of the CDR residues could substantially increase the overall solubility of the antibody, which in turn increases in the yield of the ADC preparation. The anti-MSLN ADCs attained from the methodology are highly soluble in aqueous solution with higher binding affinity, specificity and more efficient in drug delivery through receptor-mediated endocytosis in comparison with the parent antibody M9 and the other positive control antibody derived from animal immune systems. We have demonstrated in this work that the resultant anti-MSLN ADCs are highly effective in eradicating xenograft tumors in mice. The methodology provides a viable general strategy for developing ADCs, even when the antibody candidates are not exactly qualified as the targeting modules for ADC development due to the antibodies’ inferior solubility, affinity and specificity to the target antigen.

## Results

### CDR sequence preferences responsible for the antigen recognition of anti-MSLN antibody CDR-variants of M9

**Figure 1:**
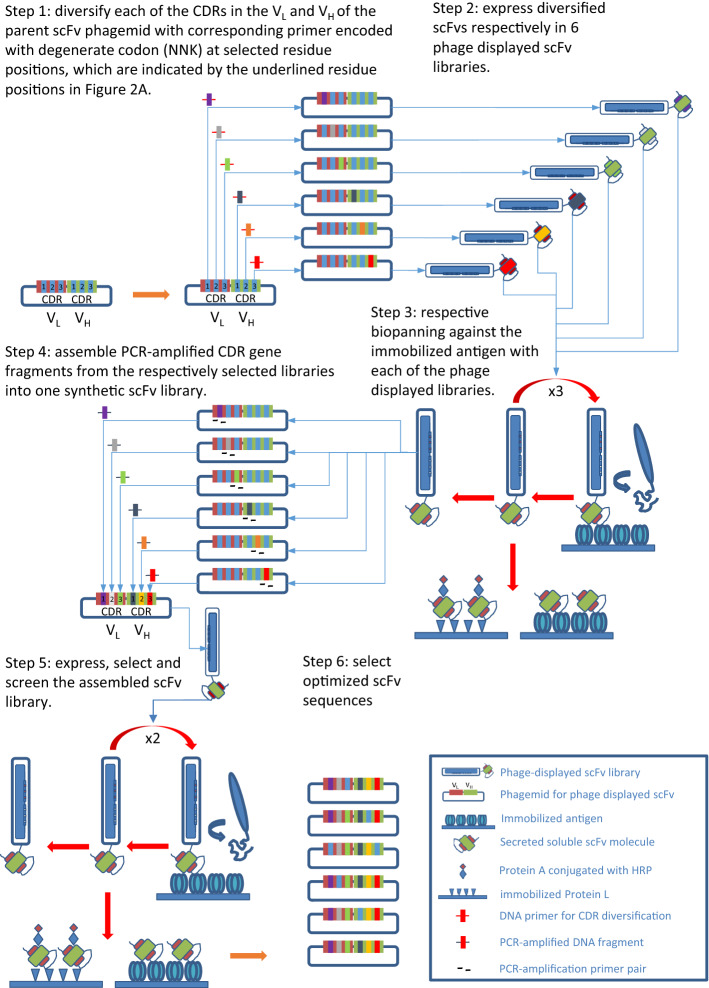
Schematic depiction of the methodology based on phage-displayed synthetic antibody libraries to explore CDR sequences of the M9-derived antibodies binding to MSLN on the same epitope as that of M9. The step-by-step procedures described in the figure are elaborated in “Methods” section. The experimental details followed the procedures previously published^25,27,33^.

**Figure 2:**
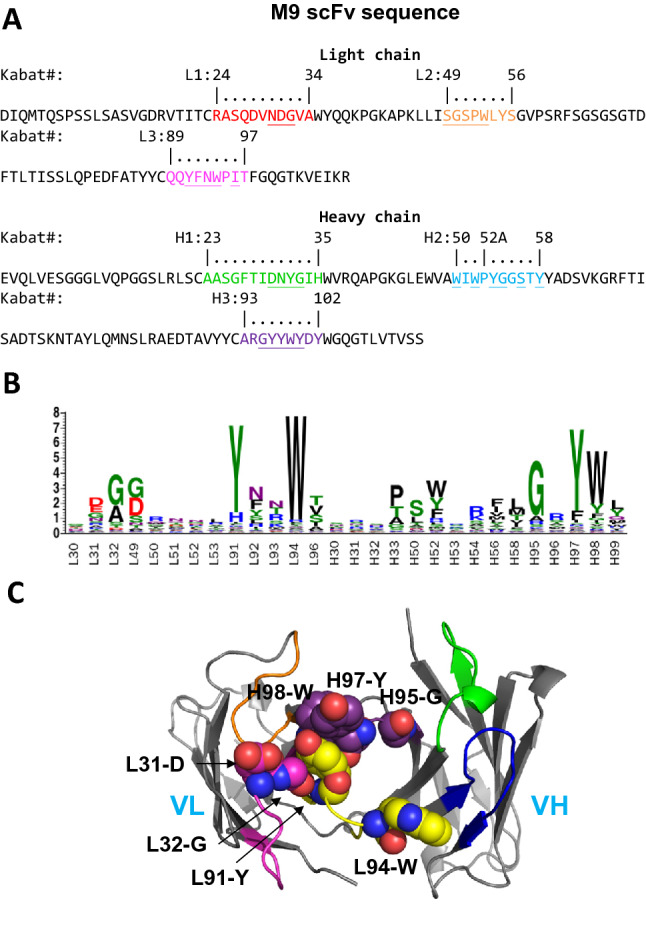
Amino acid sequences of the anti-MSLN M9 antibody variable domains and MSLN-positive scFv CDR-variants of M9 selected from the phage-displayed synthetic antibody libraries based on the methodology depicted in Fig. 1. (**A**) Sequences of the M9 light chain and heavy chain variable domain are labeled with Kabat numbering. The colored regions are the CDRs defined by North et al.^34^. Underlined CDR residues were diversified with degenerated codon NNK in constructing the phage-displayed synthetic antibody libraries in Step 1 of Fig. 1. (**B**) The panel shows the sequence LOGO of the scFv CDR-variants of M9 from Step 6 of the procedure shown in Fig. 1. The scFv CDR-variant sequences are listed in Supplementary Table S1. (**C**) The M9 light chain and heavy chain scFv VL/VH variable domain structures were computationally modelled with RosettaAntibody modeling software^35^ with default parameters. Amino acid residues in van der Waals spheres are labeled according to the Kabat numbering as shown in panel (**A**). The CDR loops are colored in red, orange, yellow, green, blue and purple for L1, L2, L3, H1, H2 and H3 respectively. The secondary structures of the M9 scFv are shown by grey arrow ribbon connected with cylindrical loops.

Anti-MSLN antibody M9 was attained with phage-displayed synthetic antibody libraries previously described by Jian et al.^25^ We developed a methodology to optimize M9 CDR sequences for anti-MSLN ADCs. The methodology is schematically depicted in Fig. 1 and the step-by-step procedures are described in “Methods” section. The scFv sequence of M9 is shown in Fig. 2A, where the CDR sequences are highlighted in colors. The MSLN-positive scFv CDR-variants of M9 from the output libraries of the phage display selections in step 6 of Fig. 1 were used to derive the CDR sequence preference profile of M9 (Fig. 2B). The CDR sequences of the MSLN-positive scFv CDR-variants of M9, which are defined by the positive binding of the soluble scFv to MSLN, Protein L and Protein A as indicated in Step 5 of Fig. 1 (Protein L/A binding indicates proper folding of the scFv structure^31,32^), are shown in Supplementary Table S1. Figure 2B indicates that CDRL3 and CDRH3 are prominent in the sequence preference profile of M9, and hence some of the residues in these two CDRs (in particular, L91-Y and L94-W in CDRL3 and H97-Y and H98-W in CDRH3) form the functional paratope (Fig. 2C) on the scFv CDR-variants of M9^27,33^. The conservativeness in the CDR sequence preference of L3 and H3 reflects the conserved local CDR-antigen interactions, suggesting that the scFv CDR-variants of M9 from Step 6 in Fig. 1 bind to the same epitope on MSLN as that of M9^27,33^. The rest of the CDRs are relatively less conserved in sequence preferences (Fig. 2B), suggesting that these regions are in the peripheral area of the M9-MSLN functional interface, and thus are less stringent in sequence requirements of the scFv CDR-variants for MSLN recognition.

### scFv candidates for ADC development assessed with high throughput in vitro cytotoxicity and flow cytometry binding assays

We attained single clonal MSLN-positive scFv CDR-variants of M9 from Step 5–6 of Fig. 1 for further specificity and affinity assessments; the sequences of these scFv CDR-variants of M9 are shown in Supplementary Table S1 and the sequence preference profile of these variants is shown in Fig. 2B. Each of the monoclonal scFvs was secreted in the medium of each individual *E. coli* cell culture harboring the corresponding monoclonal scFv phagemid. To assess these scFvs as targeting modules for anti-MSLN ADCs, we non-covalently conjugated the soluble scFvs with AL1-RFP (Protein A-Protein L-red fluorescence protein fusion protein) and AL1-PE38KDEL (Protein A-Protein L-Pseudomonas exotoxin A) for mean fluorescence intensity (MFI)^25^ and cytotoxicity measurements^25^ respectively. Both measurements were carried out on cultured human cancer cell lines of N87 and H226, both with MSLN expressed on the cell surface. The AL1-RFP and AL1-PE38KDEL are fusion proteins with single polypeptide chain containing Protein A, Protein L and RFP or PE38KDEL respectively; Protein A and Protein L in the fusion proteins non-covalently bind to the heavy chain and light chain of the scFv respectively with 1:1 molar ratio in nM affinity^24^. The binding of Protein A and Protein L to the natively folded scFv structure does not interfere with the paratope-epitope interface of the scFv-antigen interaction^24^.

**Figure 3:**
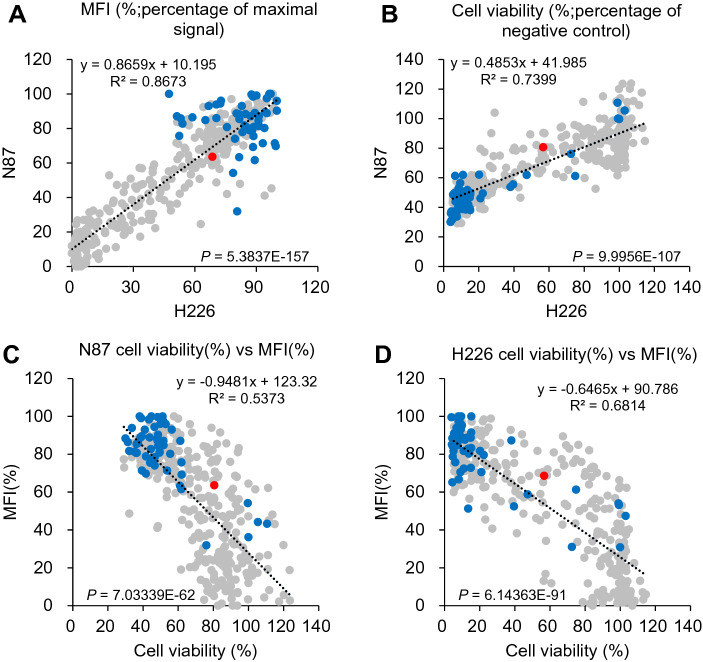
Binding and cytotoxicity characterizations of the scFv CDR-variants of M9 as targeting candidates for anti-MSLN ADCs. (**A**) Mean fluorescence intensities (MFIs) for each of the AL1-RFP-conjugated scFv CDR-variants of M9 binding to N87 and H226 cells are shown in the y-axis and x-axis respectively. The MFIs were normalized to the percentages of the corresponding maximal signal to facilitate the comparison of the binding of the scFvs to N87 and H226 cultured cells. (**B**) Percentages of cell viability of N87 and H226 cell cultures treated with each of the AL1-PE38KDEL-conjugated scFv CDR-variants of M9 are shown in the y-axis and x-axis respectively. The cell viability was normalized to the percentage of the cell viability of the corresponding negative control to facilitate the comparison of the cytotoxicities of the scFv-based immunotoxins to N87 and H226 cultured cells. The panels of (**C**) and (**D**) show the correlation of MFI (y-axis) versus cell viability (x-axis) for N87 and H226 cultured cells respectively. The data points for the M9 scFv are colored in red; the data points in grey and blue are the scFv CDR-variants of M9, for which the sequences are shown in Supplementary Table S1. The blue data points are the scFv candidates selected, in the studies followed, to be reformatted into human IgG1 framework for further characterizations as ADC candidates against MSLN. Numerical data and CDR sequence for each of the data points are shown in Supplementary Table S1.

The in vitro assessments of the selected scFv CDR-variants of M9 indicate that specific scFv-MSLN binding results in receptor mediated endocytosis of the scFv. AL1-RFP MFI measurements and AL1-PE38KDEL cytotoxicity measurements with N87 cells are positively correlated with the corresponding measurements using H226 cells with R^2^ = 0.87 and 0.74 respectively (Fig. 3A, B respectively). The high correlations indicate that these MSLN-positive scFvs bound to the MSLN expressed on the cell surface of both cell lines, and that the cytotoxicities of the scFv-AL1-PE38KDEL immunotoxins are attributed to the binding of the scFv to the cell surface MSLN, rather than non-specific cytotoxic effect independent to the scFv-MSLN binding. These two implications are further illustrated with the plots of the MFI of scFv-AL1-RFP versus the cytotoxicity of scFv-AL1-PE38KDEL in Fig. 3C for N87 and in Fig. 3D for H226 cultured cells. The negative correlations between the MSLN binding and the cell viability for the scFv CDR-variants of M9 with R^2^ = 0.54 and 0.68 respectively for N87 and H226 cells (Fig. 3C, D) are consistent with the most likely implication that the scFv-AL1-PE38KDEL cytotoxicity is due to cell surface receptor mediated endocytosis of the immunotoxin through the specific scFv-MSLN binding. The cytotoxicity of the immunotoxins on H226 cells was more potent in comparison with that on N87 cells (Fig. 3C, D), most likely due to the fact that H226 cells express more MSLN on the cell surface than N87 cells do, as judged by the higher absolute MFIs measured with the scFv-AL1-RFPs on H226 cells (Supplementary Figure S1).

### Antibody solubility in aqueous environment as a critical determinant for the antibodies as targeting modules for anti-MSLN ADCs

Based on the scFv-MSLN interaction data in Fig. 3A–D, we reformatted 55 scFv CDR-variants of M9 with human IgG1 framework for further evaluation of specificity and efficacy of these IgG1s as targeting modules for anti-MSLN ADCs. These scFvs were selected mostly with both strong cell surface MSLN binding and potent immunotoxin cytotoxicity—the data points for these selected scFvs are colored in blue in Fig. 3A–D; their CDR sequences are shown in Supplementary Table S1.

Since the solubilities of the IgG1s as ADC candidates were expected to be critical for the ADCs’ preparation and efficacy, we devised a relative hydrophilicity score (RH-score) for a query scFv CDR-variant of M9 to anticipate the solubility of the IgG1 reformatted from the query scFv in comparison with that of IgG1-M9:

1 $$ \begin{aligned} & {\text{H - score}}\left( {{\text{query}}\;{\text{scFv}}} \right) = 1 \times \left( {{\text{number}}\;{\text{of}}\;{\text{`STNQG'}}\;{\text{amino}}\;{\text{acid}}\;{\text{types}}\;{\text{in}}\;{\text{the}}\;{\text{CDRs}}\;{\text{of}}\;{\text{the}}\;{\text{query}}\;{\text{scFv}}} \right) \\ & \quad + 2 \times \left( {{\text{number}}\;{\text{of}}\;{\text{`KRHDE'}}\;{\text{amino}}\;{\text{acid}}\;{\text{types}}\;{\text{in}}\;{\text{the}}\;{\text{CDRs}}\;{\text{of}}\;{\text{the}}\;{\text{query}}\;{\text{scFv}}} \right) \\ & {\text{RH - score}}\left( {{\text{query}}\;{\text{scFv}}} \right) = {\text{H - score}}\left( {{\text{query}}\;{\text{scFv}}} \right){-}{\text{H - score}}\left( {{\text{M9}}\;{\text{scFv}}} \right) \\ \end{aligned} $$

**Figure 4:**
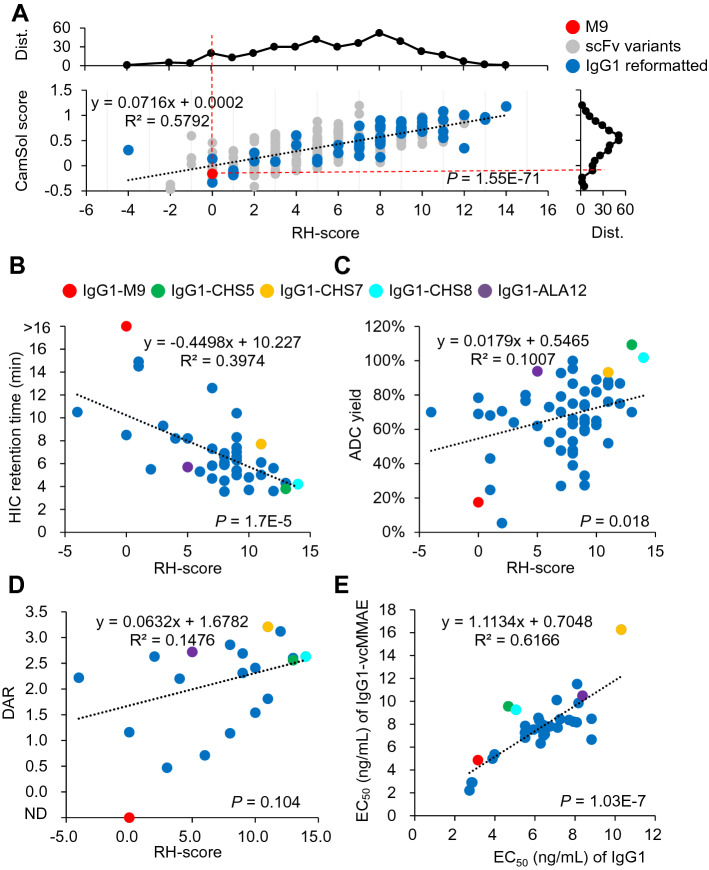
Characterization of the ADC preparations for IgG1-vcMMAE. (**A**) The grey and blue data points in the scatter plot are the CamSol scores^36^ (y-axis) plotted against the RH-scores (x-axis) for the MSLN-positive scFv CDR-variants of M9 shown in Fig. 3 (sequences are listed in Supplementary Table S1). The data point for scFv-M9 is colored in red. The data points for the 55 selected scFv CDR-variants of M9 reformatted with human IgG1 framework are colored in blue. The distribution curve above the scatter plot shows the number of scFv CDR-variant of M9 (y-axis) at each bin of the RH-score (x-axis); the distribution curve at the right-hand side of the scatter plot shows the number of scFv CDR-variant of M9 (x-axis) at each bin of the CamSol score (y-axis). (**B**) IgG1s reformatted from selected scFv CDR-variants of M9 were characterized with hydrophobic interaction chromatography (HIC). The HIC retention times of the IgG1s (y-axis) are plotted versus their corresponding RH-scores (x-axis). The R^2^ and the *P* value in this panel were calculated without including the data point for M9 colored in red. (**C**) ADC yields of the IgG1-vcMMAEs are plotted versus their corresponding RH-scores. (**D**) DARs of the IgG1-vcMMAEs are plotted versus their corresponding RH-scores. The DAR for M9-vcMMAE could not be measured with HIC, as shown in Supplementary Figure S2, and thus the datapoint (colored in red) for the DAR of M9-vcMMAE is indicated by ND in the y-axis. The R^2^ and the *P* value in this panel were calculated without including the data point for M9 colored in red. (**E**) The EC_50_’s of the IgG1-vcMMAEs binding to MSLN are plotted versus the EC_50_’s of the IgG1 binding to MSLN. In panels (**B**)–(**E**), data points for IgG1-M9, IgG1-CHS5, IgG1-CHS7, IgG1-CHS8 and IgG1-ALA12 are colored in red, green, orange, cyan and purple respectively. Numerical data and CDR sequences for each of the data points are shown in Supplementary Table S1.

The CDRs of the scFv in Eq. (1) have been defined in Fig. 2A. The RH-scores of the MSLN-positive scFv CDR-variants of M9 are plotted in Fig. 4A against the CamSol scores calculated with the corresponding scFv sequences; the CamSol scores were predicted with the CamSol computer algorithm, which has been validated, with accuracy to an extent, in predicting actual protein solubility in aqueous solution based on the protein sequence^36^. The distribution curves in Fig. 4A show that more than 90% of the MSLN-positive scFv CDR-variants of M9 have higher RH-score (the upper distribution curve in Fig. 4A) and higher predicted solubility (the right-hand side distribution curve in Fig. 4A) in comparison with those of the parent M9 scFv (red dashed lines in Fig. 4A), indicating that the optimized CDR sequences from the methodology shown in Fig. 1 for MSLN binding are more hydrophilic and predicted to be more soluble in water. The positive correlation (R^2^ = 0.58 and *P* value = 1.5 × 10^–71^) between the RH-score and the CamSol score indicates that the predicted solubility of the antibodies by CamSol is semi-quantitatively related to the number of hydrophilic/charged amino acid types in the CDRs of the scFv CDR-variants of M9, suggesting that, as expected, increasing the number of the hydrophilic/charged residues in the CDRs of an antibody is expected to increase the solubility of the antibody in aqueous environment.

The solubilities of the IgG1s reformatted from the selected scFv CDR-variants of M9 were measured by the retention time of HIC (hydrophobic interaction chromatography), which is a hydrophobicity indicator for the analyte protein, and is expected to be negatively correlated with the protein solubility in water^37^. The HIC retention times are plotted against the RH-scores of the reformatted IgG1s in Fig. 4B. The HIC retention time is negatively correlated with the RH-score with R^2^ = 0.40 (*P* value = 1.7 × 10^–5^) (Fig. 4B), in agreement with the expectation that the IgG1s with increasingly large RH-score are increasingly more hydrophilic and hence are predicted to be more soluble in aqueous solution in comparison with IgG1-M9 (Fig. 4B).

IgG1s were conjugated with vcMMAE (monomethyl auristatin E linked to the IgG1 via valine-citrulline dipeptide cathepsin-cleavable linker) through the cysteines of the reduced disulfide bonds on the IgG1s following the standard procedure^29^. The DARs (drug–antibody ratios) were measured with hydrophobic interaction chromatography (HIC) (Supplementary Table S1). None of the ADCs, for which the DARs were measured successfully, aggregated. The HIC data of a representative subset of IgG1s and their corresponding IgG1-vcMMAEs are shown in Supplementary Figure S2, where the HIC analyses of the IgG1-vcMMAEs on a butyl-NPR column yielded peaks corresponding to different vcMMAE:IgG1 ratios, and the distributions of the peaks were used to calculate the DARs for the IgG1-vcMMAEs.

Antibody hydrophilicity promotes ADC yield and DAR for vcMMAE conjugation to IgG1s. The ADC yield increases with increasing RH-score, but the positive correlation is weak with R^2^ = 0.10 (*P* value = 0.018) (Fig. 4C). The DAR (drug–antibody ratio) of the ADCs also increases with increasing RH-score with R^2^ = 0.15 (*P* value = 0.10) (Fig. 4D). Both results suggest that the hydrophilicity of the IgG1s facilitates the vcMMAE-to-IgG1 conjugation, albeit with weak positive correlation. Moreover, as shown in Fig. 4E, the half maximal effective concentrations for MSLN binding (MSLN-EC_50_’s) of the IgG1-vcMMAEs versus the MSLN-EC_50_’s of the IgG1s are plotted with the linear correlation of slope = 1.1 and R^2^ = 0.62 (*P* value = 1.0 × 10^–7^). The correlation indicates that the conjugation of vcMMAE to the cysteines of the reduced disulfide bonds on the IgG1 has little impact on the binding of the IgG1 to MSLN.

Together, as shown in Fig. 4 and Supplementary Figure S2, IgG1-M9 is not feasible as an ADC candidate because of the low ADC yield and DAR due to the overly hydrophobic CDRs (Supplementary Figure S2 and the red data points in Fig. 4A–D). By contrast, the IgG1s reformatted from the scFvs selected with the binding and cytotoxicity characterizations as shown in Fig. 3 are likely to be feasible ADC candidates. These IgG1s conjugated with the hydrophobic drug vcMMAE with higher ADC yield and DAR in comparison with those of M9 (Supplementary Figure S2, Supplementary Table S1 and Fig. 4). The feasibility of these IgG1s as candidates for ADC development is attributed to the hydrophilic/charged amino acids encoded in the CDR-variant sequences, as indicated by the relatively high RH-score in comparison with that of M9 (Fig. 4B–D and Supplementary Table S1).

### Potencies of the IgG1s reformatted from the selected scFvs as targeting modules for PE38-based immunotoxins and ADCs conjugated with vcMMAE

**Figure 5:**
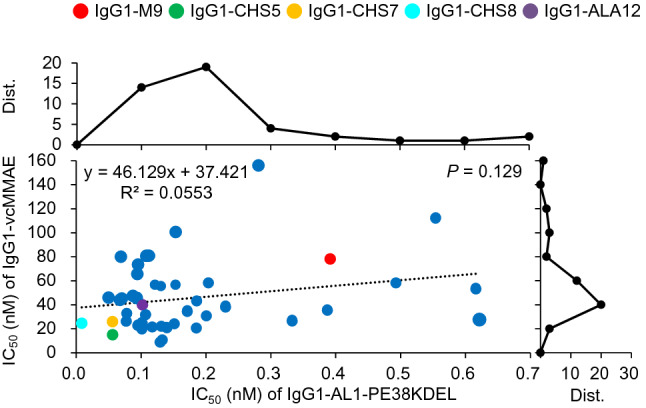
IC_50_’s of IgG1-vcMMAEs and IgG1-AL1-PE38KDEL. The IC_50_’s (nM) of the IgG1-vcMMAEs (y-axis) with the IgG1s reformatted from the selected subset of the scFv CDR-variants of M9 are plotted versus the IC_50_’s (nM) of the IgG1-AL1-PE38KDELs (x-axis). Cytotoxicity data measured with N87 cultured cells were used to calculated the IC_50_’s of these immunotoxins and ADCs. The distribution curve above the scatter plot shows the number of IgG1-AL1-PE38KDEL (y-axis) at each bin of the IC_50_ (nM) (x-axis); the distribution curve at the right-hand side of the scatter plot shows the number of IgG1-vcMMAE (x-axis) at each bin of the IC_50_ (nM) (y-axis). The data points for IgG1-M9, IgG1-CHS5, IgG1-CHS7, IgG1-CHS8 and IgG1-ALA12 are colored in red, green, orange, cyan and purple respectively. Numerical data and CDR sequences for each of the data points are shown in Supplementary Table S1.

The PE38KDEL-based immunotoxins and the vcMMAE-conjugated ADCs based on the IgG1s in Fig. 4 reformatted from the selected scFv CDR-variants of M9 have potent cytotoxicity in vitro against N87 cultured cells (Fig. 5). As expected, the half maximal inhibitory concentrations (IC_50_’s) of the IgG1-AL1-PE38KDELs are clustered to the optimal value (about 0.1–0.2 nM as shown in upper distribution curve in Fig. 5) because these IgG1s were reformatted from the scFv CDR-variants of M9 selected with potent scFv-AL1-PE38KDEL cytotoxicity (Fig. 3). Similarly, the IC_50_’s of IgG1-vcMMAEs are clustered between 20 and 80 nM (the right-hand side distribution curve in Fig. 5), indicating that the same set of IgG1s are also effective as the targeting modules for the vcMMAE-based ADCs against N87 cultured cells in vitro. Nevertheless, the correlation between the two sets of IC_50_’s is insignificant (R^2^ = 0.056 and *P* value = 0.13; Fig. 5), indicating that the cytotoxic mechanisms for the IgG1-AL1-PE38KDEL and IgG1-vcMMAE are not quantitatively related and hence the potency of the vcMMAE-based ADCs could only be qualitatively inferred from the cytotoxicity of the PE38KDEL-based immunotoxins. Although the IC_50_’s of the IgG1-vcMMAEs are expected to be related to the corresponding DARs of the ADCs, the correlation of DAR versus IC_50_ is insignificant, as shown in Supplementary Figure S3, indicating that the IC_50_’s of the ADCs are dependent on other factors, such as IgG1-MSLN interaction affinity, in addition to the DARs.

Although the IC_50_ measurements indicated that the IgG1-AL1-PE38KDEL immunotoxins were about 1–2 orders of magnitude more potent than the IgG1-vcMMAEs (Fig. 5), the systemic toxicities of these immunotoxins in animal disease models had discouraged further development of these immunotoxins as therapeutics against tumors^29^. We thus focused only on a few selected IgG1-vcMMAEs for in vivo validation as cancer therapeutics in the following sections. The data associated with these selected IgG1s are shown in Figs. 4 and 5 for IgG1-vcMMAE characterization and in Supplementary Figure S2 for HIC analyses. The data points associated with these IgG1s are colored in green (IgG1-CHS5), orange (IgG1-CHS7), cyan (IgG1-CHS8) and purple (IgG1-ALA12) in the Figures and Tables. These IgG1s were selected because of their high ADC potency (Fig. 5), high ADC yield (Fig. 4C) and DAR (Fig. 4D), likely due to the high hydrophilicity of the CDRs in the IgG1s, as reflected by the high RH-scores and short HIC retention times for these IgG1s (Fig. 4B and Supplementary Figure S2). The high affinity and specificity of these IgG1 are attributed to the highly conserved aromatic residues in the CDRs of the variable domains, for which the model structures and the conserved CDR aromatic residues are shown in Supplementary Figure S4.

### Specificity of the M9-derived MSLN-positive IgG1s in delivering cytotoxic payload through binding to the cell surface MSLN

**Figure 6:**
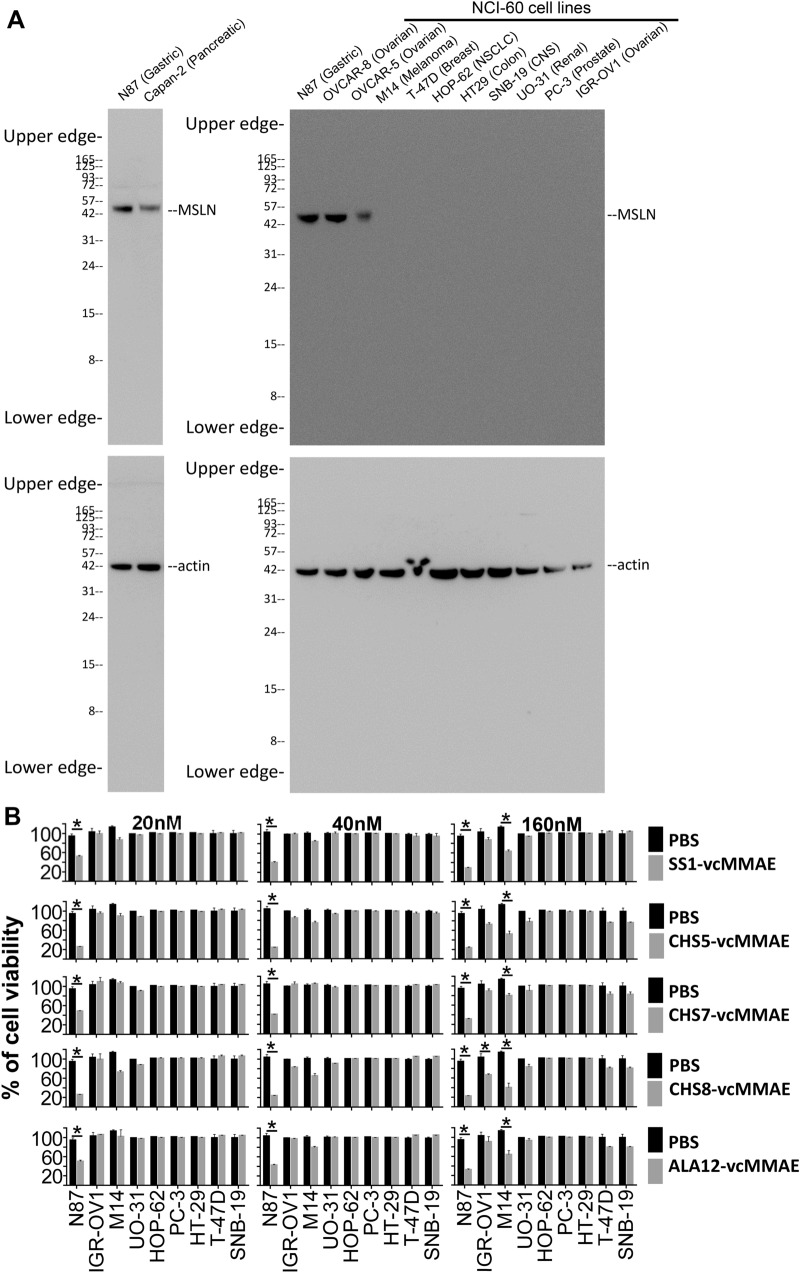
Specificity of the IgG1-vcMMAEs in targeting MSLN on cancer cell surface. (**A**) The full-length Western blots show mesothelin expression levels of the positive control cells: N87 (gastric), Capan-2 (pancreatic), OVCAR-8 (ovarian) and OVCAR-5 (ovarian) and the negative control cells: M14 (melanoma), T-47D (breast), HOP-62 (lung), HT29 (colon), SNB-19 (CNS), UO-31 (renal), PC-3 (prostate) and IGR-OV1 (ovarian). The MSLN signals in the upper panels of this figure were taken from PVDF membranes first probed with anti-MSLN antibody, and the actin signals in the lower panels of this figure were taken with anti-actin antibody as the internal controls after stripping the membrane. These full-length blot images are original without brightness or contrast adjustment; Supplementary Figure S5 shows these blot images in different brightness and contract adjustments for comparisons. (**B**) Cell viabilities (y-axis) are shown for each of the negative and positive control cells (x-axis) for the 4 selected IgG1-vcMMAEs and 1 positive control SS1-vcMMAE, for which the antibody sequence has been derived from Chowdhury et al.^38^ The cell viability measurements were repeated three times and the standard deviations are shown by the error bars. Two-tailed paired Student t-test *P* values indicate statistical significance (*P < 0.05).

**Figure 7:**
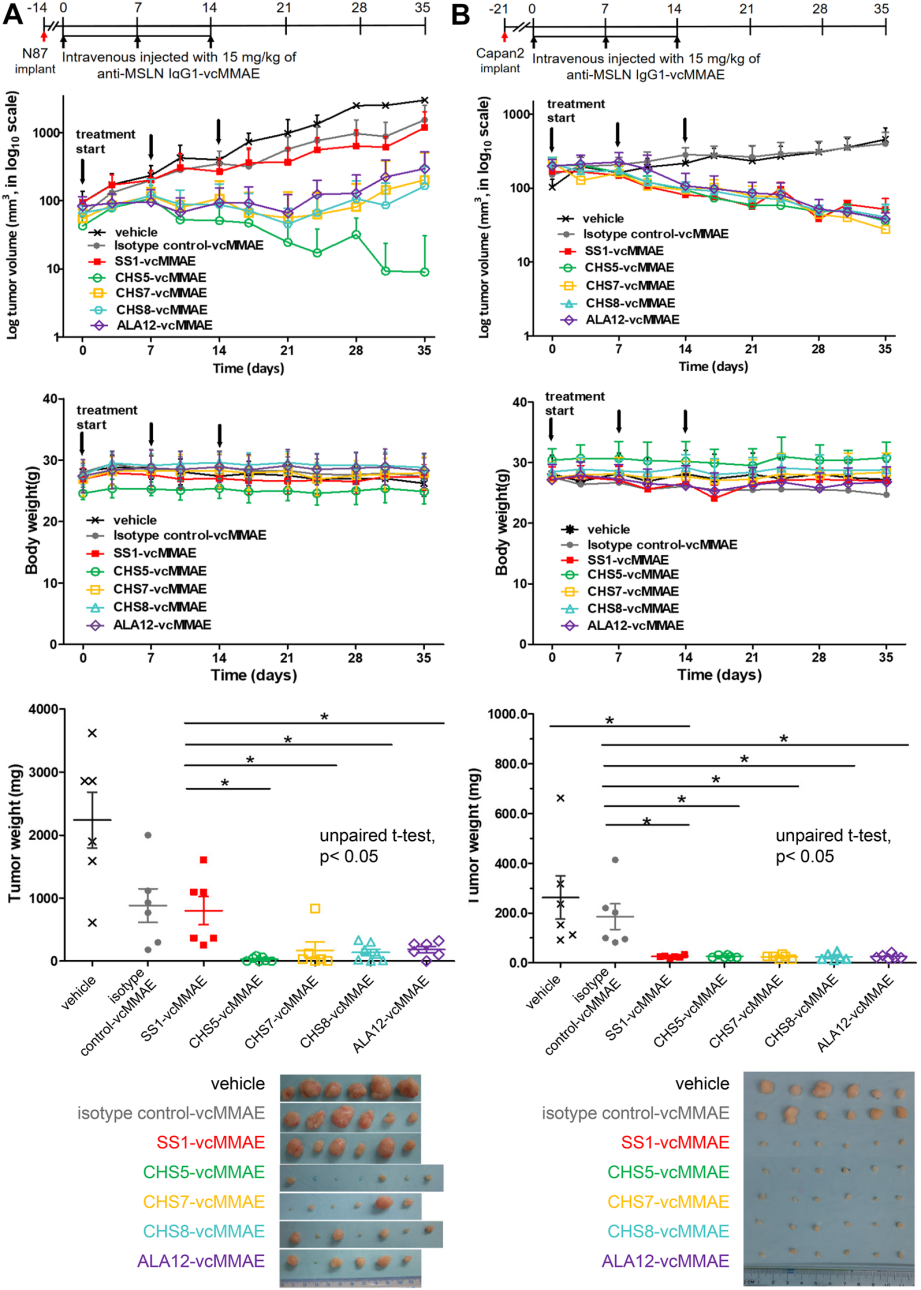
Treatments of N87 and Capan-2 xenograft mouse models with anti-MSLN IgG1-vcMMAEs. Four IgG1-vcMMAEs (CHS5-vcMMAE, CHS7-vcMMAE, CHS8-vcMMAE, and ALA12-vcMMAE) of the experimental ADC group were used for the in vivo treatments of the N87 (**A**) and Capan-2 (**B**) xenograft mouse models and the treatment results are compared with those of the positive ADC control (SS1-vcMMAE), isotype ADC control (S40-vcMMAE) and vehicle control. For both (**A**) and (**B**), the first panel from the top shows the treatment schedule, where the anti-MSLN IgG1-vcMMAE treatments were carried out on the xenograft mouse models randomly assigned into 7 groups (n = 6–7 per group) by treating (intravenous injection) with 15 mg/kg of respective anti-MSLN IgG1-vcMMAE at 0, 7 and 14 days. The second and the third panels from the top show the tumor volume and body weight of the xenograft mice continuously measured until day 35 post treatment. Two-tailed paired Student t-test *P* values indicate statistical significance (*P < 0.05). The fourth panel from the top shows the endpoint tumor weight at day 35 for each of experimental subjects plotted for each treatment group; the excised tumors used for tumor weight measurements are shown in the photographs below this panel.

**Figure 8:**
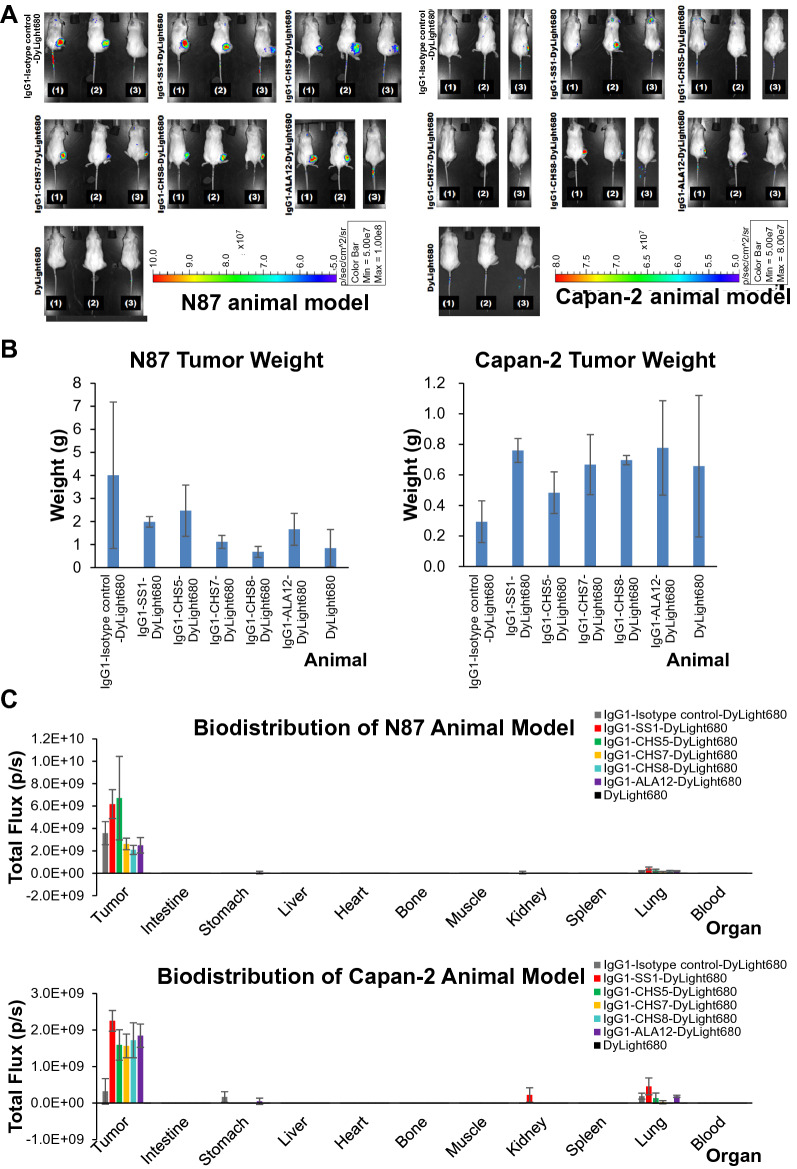
Bio-distributions of the anti-MSLN IgG1s in N87 and Capan-2 xenograft models. (**A**) DyLight 680-labeled anti-MSLN IgG1s of the experimental group (IgG1-CHS5-DyLight 680, IgG1-CHS7-DyLight 680, IgG1-CHS8-DyLight 680 and IgG1-ALA12-DyLight 680), along with the isotype control (IgG1-S40-DyLight 680), positive control (IgG1-SS1-DyLight 680) and negative control (DyLight 680 only), were injected into N87/Capan-2 tumor-bearing mice and imaged at 24 h post-injection (0.5 nmol, 150 μL per injection) with IVIS. (**B**) The mean value and standard deviation of tumor weight for each of the experimental groups were calculated with the data collected from the three mice in the corresponding experimental group as shown in (**A**). (**C**) Ex vivo quantified bio-distributions of DyLight 680-labeled IgG1 in N87/Capan-2 tumor-bearing mice at 24 h post-injection are shown in the y-axis for the tumors and organs excised from the mice (x-axis). The mean values and standard deviations of bio-distributions were calculated with three mice in each of the experimental groups. The ex vivo images of the tumors and organs with IVIS are shown in Supplementary Figure S6.

We tested the cytotoxic specificities of the 4 selected IgG1-vcMMAEs as described above: CHS5-vcMMAE, CHS7-vcMMAE, CHS8-vcMMAE and ALA12-vcMMAE and compared the specificities with that of the positive control SS1-vcMMAE, for which the anti-MSLN antibody has been described in Chowdhury et al.^38^ Although there are many anti-MSLN antibodies known in public domain^1,39^, the reason for our choice of using SS1 anti-MSLN antibody as positive control antibody is that SS1 has been documented in many publications, including the high-resolution structure deposited in PDB (code 4F3F for Amatuximab)^40^. Moreover, SS1-based therapeutics have been registered in human trials (Amatuximab and SS1P) with public information available for reproducing the antibody SS1 in the same IgG1 framework for side-by-side comparisons with the ADCs of this work in terms of vcMMAE conjugation (Supplementary Figure S2), cell-based cytotoxicity measurements (Fig. 6), in vivo efficacy (Fig. 7) and biodistribution of the vcMMAE-based ADCs (Fig. 8).

We selected, with the CellMiner^41^ webserver, a panel of 8 NCI-60 cell lines^42^ of different organ origin without MSLN expression and verified the absence of MSLN expression in these culture cells in comparison with 4 MSLN-positive control cells (Fig. 6A). We then measured the cytotoxicity of the 5 IgG1-vcMMAEs against the cell lines with/without MSLN expression in the presence of the ADC concentration of one, two, and eightfolds of the average IC_50_ (Fig. 6B). Other than the positive ADC cytotoxicity on the positive control N87 cells, the results in Fig. 6B show that significant cytotoxicity was not found for the MSLN-negative cells of different organ origin at ADC concentrations below twofolds of the average IC_50_. Although, two NCI-60 cell lines (M14 and, to a lesser extent, IGR-OV1) were subject to ADC cytotoxicity at the highest ADC concentration (Fig. 6B), the 5 IgG1-vcMMAEs had similar specificity patterns in terms of cytotoxicity against the representative panel of cell lines, suggesting a possibility that the M14 cells could express minor amount of MSLN, of which the expression level was below the detection limit of the Western Blot shown in Fig. 6A. The results implied that the off-target toxicity in in vivo treatments of MSLN-positive tumors with these IgG1-vcMMAEs would be unlikely, because the efficacies of the ADCs tested in Fig. 6 were clearly associated only with the specific targeting of the cell surface MSLN by the antibodies in the ADCs.

### In vivo treatments of xenograft tumors in mouse disease models with the anti-MSLN IgG1-vcMMAEs

We treated xenograft N87 (human gastric) and Capan-2 (human pancreatic) tumors in mice with the four IgG1-vcMMAEs (CHS5-vcMMAE, CHS7-vcMMAE, CHS8-vcMMAE and ALA12-vcMMAE) in the experimental ADC group and compared the results with those of the positive control treatment with SS1-vcMMAE and those of two negative control experiments with the isotype ADC and vehicle treatments. N87 and Capan-2 cancer models were selected because both are important human cancers with limited treatment options and both cancer cells express MSLN on the cell surface (Fig. 6A). Figure 7A and B show the in vivo treatment results on N87 and Capan-2 xenograft tumors in mice respectively. Both experiments indicate that the four anti-MSLN IgG1-vcMMAEs in the experimental ADC group were comparable or better in treating the N87 and Capan-2 mouse xenograft disease models in comparison with the positive control ADC (SS1-vcMMAE), and the endpoint results of almost complete eradication of xenograft tumors in several of the treatments clearly demonstrated the superior efficacies of the IgG1-vcMMAEs in the experimental ADC group comparing with those of the negative control treatments (Fig. 7).

Bio-distributions of the IgG1s in xenograft models one day after the treatments showed that the control and experimental ADCs tested in the in vivo treatments above were overwhelmingly concentrated in the xenograft tumors. The 4 experimental IgG1s, along with the positive and isotype control IgG1s, were conjugated with fluorescence dye and the in vivo fluorescence imaging with these IgG1-dye conjugates indicated that the IgG1s were locally concentrated in the N87/Capan-2 xenograft tumors one day after the administration of the IgG1-dye conjugates to the xenograft tumor mice (Fig. 8A and Supplementary Figure S6). Quantitative ex vivo measurements of the bio-distributions showed that all the IgG1s targeted the N87/Capan-2 tumors with high local concentration and low off-target propensity to all the organs (Fig. 8C and Supplementary Figure S6), although insignificantly minor distributions of the IgG1s in the lung of the mouse disease models were also found. The tumor-heavy bio-distributions of the IgG1s (Fig. 8C and Supplementary Figure S6) agree with these IgG1s’ MSLN-specific targeting capabilities demonstrated in Fig. 6.

The serum biochemical parameters in N87/Capan-2 xenograft mice at the treatment endpoint (three weeks after the treatments with the IgG1-vcMMAEs) showed that the in vivo treatments with these IgG1-vcMMAEs were not accompanied with long-lasting adverse side effects due to toxicities (Supplementary Tables S2 and S3).

Less effective treatments with partial tumor eradication in N87 disease models by the positive control and some of the IgG1-vcMMAEs in the experimental ADC group (Fig. 7A) could result from the extreme tumor burden of the N87 xenograft mice during the ADC treatments. In comparison with the Capan-2 tumors with relatively less aggressive growth rate (Figs. 7, 8 and Supplementary Figure S6), the highly aggressive growth of the N87 xenograft tumors in mice could also account for the intake of the isotype control IgG1 in the N87 tumor (Fig. 8 and Supplementary Figure S6), explaining the partial effectiveness for the isotype control ADC in treating the N87 tumor disease models (Fig. 7A).

Overall, the 4 IgG1-vcMMAEs in the experimental ADC group are effective ADC therapeutics with non-detectable off-target toxicity in treating N87 and Capan-2 xenograft tumors in mice. In addition, the in vivo treatments with CHS5-vcMMAE are of particular interest because of its superior efficacies in tumor eradication in both tumor disease models. Although IgG1-CHS5’s affinity to MSLN is not the highest among the IgG1s in the experimental ADC group (Fig. 4E) and the DAR of CHS5-vcMMAE (2.56) is the lowest among the ADCs used in the in vivo treatments, its hydrophilicity (Fig. 4B), ADC yield (Fig. 4C), in vitro cytotoxicity (Fig. 5), and cell surface MSLN-targeting specificity (Fig. 6) are superior to an extent among the IgG1s in the experimental ADC group. These results highlight the importance of the collection of the characterizations shown in these Figures in determining the efficacy and specificity of the candidate ADCs in the in vivo treatments leading to tumor eradication.

## Discussion

The methodology described in this work has been demonstrated to produce highly potent anti-MSLN ADCs in comparison with that of the parent antibody M9. With the phage-displayed synthetic antibody libraries derived from the parent M9 antibody, we used the methodology to explore CDR sequences of the M9-derived antibodies binding to MSLN on the same epitope as that of M9. The optimization of the antibody CDR sequences as targeting modules for the ADCs against MSLN-positive cancer disease models is attributed to the following progressive selection criteria: (1) Protein A and Protein L binding to assure the proper folding of the scFvs; (2) affinity of scFvs to immobilized recombinant MSLN measured with ELISA; (3) affinity screening with flow cytometry of the scFvs to cell surface expressed MSLN; (4) functional screening of the cytotoxicity of the PE38-based immunotoxins with the scFvs as the targeting modules; (5) expression efficiency of the IgG1 reformatted from the selected scFvs; (6) measurement of hydrophobicity of the IgG1s with HIC; (7) ADC preparation yield of the IgG1s conjugated with vcMMAE; (8) EC_50_ of the IgG1s and IgG1-vcMMAEs binding to MSLN; (9) IC_50_’s of the ADCs measured with MSLN-positive culture cells; (10) specificity of the IgG1s measured with off-target toxicities of the corresponding ADCs against a panel of cultured human cancer cells from diverse organ origin; (11) in vivo efficacies of the ADCs in treating xenograft tumors in mice; (12) in vivo bio-distribution of the IgG1s in terms of targeting specificity of the antibodies against the cancer-associated antigen on the cancer cell surfaces. The results of the in vivo treatment efficacies of the ADCs with the selected IgG1s demonstrated that the collection of these characterizations led to the antibodies with higher efficacies in drug delivery.

The optimization of scFv CDR-variants of M9 binding to MSLN produced a large number of M9-derived MSLN-positive scFv sequences. The CDR sequence preference profile of these scFvs reveals the roles played by the paratope residues in the CDRs in recognizing the antigen and defines the functional paratope on the M9-derived scFvs in the absence of the antibody–antigen complex structural information. From the sequence preference profiles, the CDRH3 and CDRL3 (in particular, H97-Y and H98-W in CDRH3, and L91-Y and L94-W in CDRL3) form the structurally contiguous paratope on the M9-derived scFvs with the indispensable aromatic residues^43^. The peripheral paratope residues, which are not in direct contact with the antigen, prefer diverse hydrophilic residues^27,33^ that could enhance the affinity and specificity between the antibody and the antigen. In particular, the CDRL2, CDRH1 and CDRH2 are in the peripheral area of the CDR-MSLN contact interface and thus are less stringent in sequence requirements for MSLN recognition. Hydrophilic residues in these regions not only could optimize the antibody–antigen interaction affinity and specificity through direct and water-mediated hydrogen bonding, the hydrophilic residues also increase the overall hydrophilicity of the antibodies by interacting with the bulk aqueous environment, facilitating the IgG1-vcMMAE conjugation with higher ADC yield and DAR. Together, the efficacies of the ADCs with high specificity in targeting cell surface MSLN and high potency in delivering cytotoxic payload are attributed to the conservation of the aromatic residues in the functional paratope facilitated with the hydrophilic residues in the peripheral structural paratope. In addition, the hydrophilicity of the CDRs facilitates the IgG1-vcMMAE production, leading to ADCs with superior efficacies in treating the N87 and Capan-2 xenograft tumors in mice with the endpoint results of almost completely eradicated xenograft tumors.

## Conclusion

The optimization methodology developed in this work is capable of improving (1) the antibody–drug conjugates with sufficient yield; (2) affinity and specificity of the ADCs binding to the target antigen on the biologically relevant epitope; (3) effective cytotoxic payload delivery to the cytoplasm of the target cells without off-target toxicity. All these optimizations increase the efficacy of the ADCs in treating cancers in vivo. The methodology provides a viable general strategy for developing ADCs, starting from antibodies that are not necessarily qualified as the targeting modules for ADC development due to the antibodies’ inferior solubility or affinity/specificity to the target antigen.

## Methods

### Methodology for optimizing CDR sequences of antibodies derived from a parent antibody

As shown schematically in Fig. 1, with M9 scFv as the parent template (sequence shown in Fig. 2A), we respectively constructed six synthetic scFv libraries, each of which contained degenerate codons (NNK) to diversify selected residue positions (marked in Fig. 2A) in only one M9 CDR while leaving the rest of the M9 template sequence unchanged^25,28^ (Fig. 1; Step 1). These synthetic antibody libraries were individually expressed with the M13 phage display system (Fig. 1; Step 2) and the phage-displayed scFv libraries were respectively used as input for phage display selection against immobilized MSLN (Fig. 1; Step 3). While the binding mode of the selected scFv CDR-variants of M9 to MSLN remained locked by the constant CDRs from the M9 template, the selected sequences of the diversified CDR were expected to further enhance the local interactions of the corresponding CDR with MSLN, as we have demonstrated in previous works^27,33^. Following three rounds of phage display selection on MSLN-binding with each of the six phage-displayed scFv libraries, scFvs that folded properly (with positive binding to both Protein A and Protein L) and bound to MSLN were selected and sequenced for CDR sequence analysis (Fig. 1; Step 3). After the confirmation of the sequence analysis, we respectively PCR-amplified the degenerate codon-diversified CDR from the corresponding output library of the phage display selections (Fig. 1; Step 4); the PCR-amplification primer pairs were designed with the M9 template with overlaps in the way such that another round of PCR-amplification of the mixture of the six PCR products with a pair of primers designed with the M9 template completed the scFv library on the basis of the M9 template and with the CDR sequences optimally selected to enhance local interactions between the CDRs and the M9 epitope on MSLN (Fig. 1; Step 4). This reassembled library was again expressed with the M13 phage display system and used as input for two rounds of phage display selection against MSLN (Fig. 1; Step 5). Single colonies of *E. coli* harboring individual scFv phagemid from the output libraries were cultured for soluble scFv screening and characterizations (Fig. 1; Steps 5 and 6). The scFv CDR-variants of M9 that bound to Protein A, Protein L and MSLN were selected and sequenced (Fig. 1; Step 6) (sequences shown in Supplementary Table S1).

### Guidelines for in vivo experiments involving mouse disease models

All mouse experiments shown in Figs. 7 and 8 were conducted according to relevant guidelines and experimental protocols approved by the Institutional Animal Care and Utilization Committee (IACUC) of Academia Sinica (Protocol ID: 18–07-1215). In addition, the studies involving animal disease models were carried out in compliance with the ARRIVE (http://www.nc3rs.org.uk/page.asp?id=1357**)** guidelines with the experimental details described in respective experimental procedures in Supplementary Methods and in the legends of Figs. 7 and 8. At the in vivo xenograft mouse model treatment endpoints (Fig. 7) and in the ex vivo bio-distribution studies (Fig. 8), mouse euthanasia was carried out after inhalation anesthesia gas (Isoflurane), followed by cervical dislocation. The euthanasia procedure follows the AVMA guidelines for the Euthanasia of Animals (2020 version).

All the experimental and computational technical details in this work have been published previously^24–29,33,44^ and can be found in Supplementary Methods.

## Supplementary Information

Supplementary Information.

## Abbreviations

ADC: Antibody–drug conjugate
scFv: Single chain variable domain fragment
IgG1-vcMMAE: Monomethyl auristatin E linked to the IgG1 via valine–citrulline dipeptide cathepsin-cleavable linker
MSLN: Mesothelin
CDR: Complementarity determining region
AL1-RFP: Protein A-Protein L-red fluorescence protein fusion protein
AL1-PE38KDEL: Protein A-Protein L-Pseudomonas exotoxin A
MFI: Mean fluorescence intensity
HIC: Hydrophobic interaction chromatography
DAR: Drug–antibody ratio
