# Supplementary material for: Eradicating mesothelin-positive human gastric and pancreatic tumors in xenograft models with optimized anti-mesothelin antibody–drug conjugates from synthetic antibody libraries
Source: Sci Rep. 2021 Jul 29;11:15430. doi: 10.1038/s41598-021-94902-1 (PMC8322431; doi:10.1038/s41598-021-94902-1)
Supplement: Supplementary file 1 — Supplementary Information. [file 41598_2021_94902_MOESM1_ESM.pdf]

Supplemental Information

Eradicating mesothelin-positive human gastric and pancreatic tumors in xenograft models with optimized anti-mesothelin antibody-drug conjugates from synthetic antibody libraries

Hung-Ju Hsu[1,4], Chao-Ping Tung[1,4], Chung-Ming Yu[1,4], Chi-Yung Chen[1], Hong-Sen Chen[1], Yu-Chuan Huang[1], Pei-Hsun Tsai[1], Su-I Lin[1], Hung-Pin Peng[1], Yi-Kai Chiu[1], Yueh-Liang Tsou[1], Wei-Ying Kuo[1], Jhih-Wei Jian[1], Fei-Hung Hung[1], Chiao-Yun Hsieh[1], Michael Hsiao[1], Simon Shih-Hsien Chuang[2], Chia-Ning Shen[1], Yong Alison Wang[3], An-Suei Yang[1]

[1] Genomics Research Center, Academia Sinica, Taipei, Taiwan 115.
[2]Institute of Pharmaceutics, Development Center for Biotechnology, Taipei, Taiwan 115.
[3] Koo Foundation Sun Yat-Sen Cancer Center, Taipei, Taiwan 112.
[4] These authors contribute equally.

[*] Correspondence should be addressed to: An-Suei Yang, Genomics Research Center, Academia Sinica, 128 Academia Rd., Sec.2, Nankang Dist., Taipei, Taiwan 115. Phone: +886-2-2787-1232 email: yangas@gate.sinica.edu.tw

TABLE OF CONTENTS

I. Supplementary Methods

- ***Computational modelling of antibody variable domain structures***
- ***Computation of the LOGO plots***
- ***Cell lines***
- ***Mean fluorescence intensity (MFI) of scFv labeled with AL1-RFP on cell surface by Flowcytometry***
- ***Cytotoxicity assay for scFvs***
- ***IgG1 preparation***
- ***Hydrophobic interaction chromatography (HIC)***
- ***IgG1-vcMMAE ADC preparation and ADC yield***
- ***Drug-antibody ratio (DAR) determination with HIC***
- ***Measurements of EC_{50} with ELISA for MSLN binding***
- ***Cytotoxicity (IC_{50}) measurements for IgG1-vcMMAEs an IgG1-AL1-PE38KDEL***
- ***Western blotting***
- ***Cytotoxic specificity assay***
- ***Xenograft mouse model treatment***
- ***Maleimide-DyLight 680 conjugation***
- ***Bio-distribution determination***
- ***Ex vivo NIRF imaging***
- ***Serum biochemical analysis***

II. Supplementary Figures

Figure S1. Binding of the scFv CDR-variants of M9 to MSLN expressed on the cell surfaces of two types of cultured human cancer cells.
Figure S2. HIC characterization of IgG1-vc-MMAEs.
Figure S3. DAR plotted against IC_{50} for the anti-MSLN IgG1-vcMMAEs.
Figure S4. The scFv VL/VH variable domain structures computationally modelled using RosettaAntibody with default parameters.
Figure S5. Full-length Western blots for actin and MSLN.
Figure S6. Ex vivo NIRF imaging of excised organs of N87/Capan-2 tumor bearing mice.

III. Supplementary Tables

.

Table S1. scFv CDR-variants of M9 derived from Step 5~6 of the procedure shown in Figure 1.
Table S2. Effects of anti-MSLN IgG1-vc-MMAEs on serum biochemical parameters in N87 NOD/SCID mice.
Table S3. Effects of anti-MSLN IgG1-vcMMAEs on serum biochemical parameters in Capan-2 NOD/SCID mice.

IV. References

I. Supplementary Methods

All the experimental and computational technical details described in this section have been published previously from our lab [1-8].

Computational modelling of antibody variable domain structures – The antibody VL/VH variable domain structures were computationally modelled with RosettaAntibody modeling software[9] with default parameters.

Computation of the LOGO plots – The size d_{ji} (in half-bit unit) for amino acid type i at position j in the consensus sequence LOGOs shown in Figure 2 were calculated with the equation:

$$d_{ji} = q_{ji} I_j$$
$$I_j = \sum_{i=1}^{20} 2 q_{ji} \log_2 \frac{q_{ji}}{p_i}$$
$$q_{ji} = \frac{C_{ji} + \sqrt{M_j}\, p_i}{(M_j + \sqrt{M_j})} \qquad (1)$$

C_{ji} is the count for amino acid i at position j in M_j count of the CDR sequences containing position j; p_i is the background probability for amino acid i encoded in the NNK degenerate codon [10]; the square root of M_j in the equation is the pseudo count to prevent singularity when C_{ji} equals to zero. Equation (1) is modified after the original formulation [11].

Cell lines – Human gastric carcinoma cell line NCI-N87 (ATCC CRL-5822), human lung carcinoma cell line NCI-H226 (ATCC CRL-5826), and human pancreas adenocarcinoma cell line Capan-2 (ATCC HTB-80) were purchased from American Type Culture Collection (ATCC). The NCI-N87 and NCI-H226 cells were grown in RPMI-1640 medium (Gibco, Ref 22400-089) supplied with 10% fetal bovine serum (Gibco) and 1X Anti-anti (Gibco, Ref 15240-062) at 37°C in a humidified incubator containing 5% CO_2. Capan-2 cells were grown in McCoy's 5A medium (ATCC-20-2007) supplied with 10% fetal bovine serum (Gibco) and 1X Anti-anti (Gibco, Ref 15240-062) at 37°C in a humidified incubator containing 5% CO_2. OVCAR-8, OVCAR-5, IGR OV1, M14, UO-31, HOP-62, PC-3, HT-29, T-47D and SNB-19 cell lines were obtained from NCI-60 cell panel [12] and were cultured in RPMI 1640 medium (Gibco, Ref 22400-089) with 10% fetal bovine serum (Gibco), 2 mM L-Glutamine (Gibco, Ref 25030-081) and 1X Anti-anti (Gibco, Ref 15240-062).

Mean fluorescence intensity (MFI) of scFv labeled with AL1-RFP on cell surface by Flowcytometry – MSLN-expressing culture cells were used for scFv CDR-variant binding characterization with FACS analysis. The procedure has been published by our lab previously with minor modification [5]. First, Cells were scraped and went through strainer with 40-micron pore. About 2×10^5 cells were incubated with 100μL of scFv at 4°C for 30 min, then washed once with 0.5% FBS 1X PBS (wash buffer), mixed with 1 μg AL1-RFP in 50μL wash buffer at 4°C for 20 min, and then washed twice with wash buffer. After centrifugation and resuspension, cells were analyzed for RFP signal by FACS (BD FACS Canto Ⅱ). Data analysis were performed by FACS Diva (BD). Mean fluorescence intensity (MFI) was used to indicate affinity of scFvs in binding to

the MSLN-expressing cells.

Cytotoxicity assay for scFvs **–** The procedure to determine cytotoxicity of scFvs was previously described by Hou et al [5]. Briefly, 10^4 cells/well were seeded in 96-well plates. 0.5nM scFvs were pre-incubated with AL1-PE38KDEL at a molar ratio of 1:1 for 1 hour at room temperature so as to form non-covalently linked immunotoxins. scFv-AL1-PE38KDEL mixtures were added to cell culture without serum. After 4 hours of incubation at 37°C, the antibody toxin mixture was replaced by fresh normal medium with serum. After 4 days of culture at 37°C, the number of viable cells was quantified using WST-1 (Roche) by measuring OD450. Percentage of cell viability was calculated by the following equation: % of cell viability = OD450nm (antibody treated cells)/OD450nm (negative control cells) × 100%.

IgG1 preparation **–** Antibody IgG1s were produced by recombinant IgG vector transfection to Expi293F cells (ThermoFisher Scientific, Cat. no. A14527). Expi293F cells were kept in Expi293™ Expression Medium (ThermoFisher Scientific, Cat. no. A1435102) with vent-cap baffled flask for better activity. Transfection was performed using ExpiFectamine™ 293 Transfection Kit (ThermoFisher Scientific, Cat. no. A14525) according to the manufacturer's instructions. After 3 days of incubation, the antibodies in supernatant was purified using Protein A Resin FF (GenScript, L00464) affinity chromatography. The purity of the antibodies was analyzed by sodium dodecyl sulfate-polyacrylamide electrophoresis (SDS-PAGE), and the concentration was determined by Nanodrop ND-1000 spectrophotometer.

Hydrophobic interaction chromatography (HIC) **–** TOSOH Biosciences Butyl-NPR column was used for HIC, which was performed at room temperature. Buffer A (potassium phosphate buffer, pH 7.2, 50 mM; 1.5 M Ammonium Sulfate) and buffer B (potassium phosphate buffer, pH 7.2, 50 mM) were prepared. Protein analytes were first precipitation-tested in solution of 20%:80% buffer B:A. These protein analyte solutions were centrifuged at 13000 rpm for 3 min, and the protein analyte concentrations in the supernatants were determined with Nano-drop spectrophotometry (Thermo Scientific). Protein analytes without substantial loss of expected concentration in the supernatant were analyzed with HIC. Before sample injection, the TOSOH Biosciences Butyl-NPR column was equilibrated with 5 mL of buffer A. 100 µL of protein analyte (0.5 mg/mL in buffer 20%:80% buffer B:A) were loaded onto the column with 1 mL of injection loop washing. The linear gradient from 20%:80% buffer B:A to 90%:10% buffer B:A was completed in 13 mL of running volume. The column was then washed with 1 mL 90%:10% buffer B:A, followed by washing with 4 ml of 20%:80% buffer B:A [13].

IgG1-vcMMAE ADC preparation and ADC yield **–** The procedure has been published by our lab previously with minor modification [2]. The IgG1s were conjugated with vcMMAE (MedChem Express) through the cysteine residues on the tris(2-carboxyethyl)phosphine hydrochloride (TCEP)-reduced IgG1s. Briefly, antibody was partially reduced for 1 hour at room temperature with tris(2-carboxyethyl)phosphine (TECP, Sigma-Aldrich) at 2 equivalent of reductant-to-IgG1 molar ratio. *N*-acetylcysteine was used to quench the reaction at room temperature for 30 min. The quenched reaction mixture was desalted by gel filtration with a 5 mL desalting column (Thermo Scientific); the buffer was changed into phosphate-buffered saline (PBS) and the ADC product was concentrated by centrifugal ultrafiltration (Amicon Ultra 30K MWCO, MERCK Millipore). The ADC solutions were filtered through a 0.2 µm filter and stored at 4 °C. The ADC products

were analyzed by sodium dodecyl sulfate-polyacrylamide electrophoresis (SDS-PAGE). The ADC yield of the IgG1-vcMMAE conjugation was calculated as the percentage of the IgG1 in the output ADC product over the total input IgG1.

Drug-antibody ratio (DAR) determination with HIC – The chromatographic data of HIC were smoothed by GraphPad Prism and graphed. The areas under the curve were analyzed by Origin Pro 2019 with peak analyzer module. The DAR calculation followed the application note from Waters Corporation (Robert Birdsall, Eoin Cosgrave, Henry Shion, and Weibin Chen, 2014, Automating the Determination of Drug-to-Antibody Ratio (DAR) of Antibody Drug Conjugates (ADCs) Based on Separation by Hydrophobic Interaction Chromatography (HIC)).

Measurements of EC_{50} with ELISA for MSLN binding – For determining the EC_{50} of purified human IgG1s and IgG1-vcMMAE binding to MSLN, the ELISA assays were carried out as described previously with minor modifications [7]. Briefly, MSLN (0.3 μg per well) was coated in PBS buffer (pH 7.4) on NUNC 96-well Maxisorp immunoplates overnight at 4 °C, and blocked with 5% milk in PBST [0.1% (v/v) Tween 20] for 1 hour. In the meantime, antibodies/ADCs in PBST with 5% milk were prepared at 11 concentrations by two-fold serial dilution, and then added 100 μL diluted samples to the plate. After 1 hour of binding and washing three times with PBST, 100 μL 1:5,000 anti-human IgG horseradish peroxidase antibody was added for 1 h incubation. After washing three times with PBST and twice with PBS buffer, the plate was developed for 3 min with 3,3',5,5'-tetramethyl-benzidine peroxidase substrate (TMB substrate, Kirkegaard & Perry Laboratories), quenched with 1.0 M HCl and read spectrophotometrically at 450 nm. The EC_{50} (nM) was calculated according to the Stewart and Watson method [14].

Cytotoxicity (IC_{50}) measurements for IgG1-vcMMAEs an IgG1-AL1-PE38KDEL – N87 cells (1×10^4) were seeded in 96-well plates for the IC_{50} measurements of cell viability. For IgG1-AL1-PE38KDEL, IgGs were pre-incubated with AL1-PE38KDEL at a molar ratio of 1:2 for IgG1:AL1-PE38KDEL for 1 hour at room temperature. This procedure allows the formation of non-covalently linked immunotoxins. IgG1-AL1-PE38KDEL mixtures or purified IgG1-vcMMAE solutions were added to culture medium without serum. After incubation at 37 °C for 16 hours, the medium was replaced by fresh normal medium with serum and the cytotoxicity was assessed using the WST-1 reagent (Roche) after 72 hours of incubation at 37 °C. Values were normalized by corresponding PBS-treated control cells as 100% viability and IC_{50} values were calculated by GraphPad Prism 6.0.

Western blotting – The cells were lysed in 1X RIPA buffer IV (Bio Basic) containing protease inhibitor cocktail tablet (Sigma). Equal amounts of proteins were resolved by NuPAGEBis-Tris gel (Invitrogen). The proteins were transferred from the gels to PVDF membranes, which were then probed with anti-MSLN antibody (Cell Signaling, #99966), followed by horseradish peroxidase (HRP)-conjugated secondary antibody, and visualized using chemiluminescent HRP substrate (Thermo) and ImageQuant LAS4000 (GE Healthcare). After imaging, the membrane was stripped by Western blot stripping buffer (Prod # 46430, Thermo scientific) for 10 minutes to remove the original signal and then the expression of actin was determined by HRP-labeled ACTB monoclonal antibody (Proteintech Group) for internal control.

Cytotoxic specificity assay – Approximately 2×10^4 cells of N87, IGR-OV1, M14, UO-31, HOP-

62, PC-3, HT-29, T-47D and SNB-19 cell lines were seeded in 96-well plates for each well. Different concentrations of IgG1-vcMMAE ADCs were directly added to the 10% FBS culture medium. 4 days after the ADC treatment, 10 µL of WST-1 solution (Roche) was added to each well. After 5 hours incubation at 37°C, absorbance at 450nm was determined using a Spectrofluorometer (icon Wallac 1420 Workatation). The percentage of cell viability was quantified by calculating the ratio below: % of cell viability = OD 450nm (ADC treated cells)/ OD 450nm (negative control cells) × 100%.

Xenograft mouse model treatment – All mouse experiments were conducted according to relevant guidelines and experimental protocols approved by the Institutional Animal Care and Utilization Committee (IACUC) of Academia Sinica (Protocol ID: 18-07-1215). 8-week-old male NOD/SCID mice (BioLASCO Taiwan Co. Ltd) were subcutaneously injected with tumor cells. Each mouse was implanted with 1 x 10^6 N87 cells or 3 x 10^6 Capan-2 cells and treated with anti-MSLN IgG1-vcMMAEs post 14 days or 21 days, respectively. When the tumors reached suitable tumor size of 80-100 mm^3, the mice were randomly assigned into control and treatment groups and dosing was started. Anti-MSLN IgG1-vcMMAEs (15 mg/kg) were intravenous injected into tail vein once a week for a total of three doses. Tumor volume and body weight of each xenograft mouse were continuously measured until day 35 post treatment of anti-MSLN IgG1-vcMMAEs. Endpoint tumor volume at day 35 for each of experimental subjects are plotted for each treatment group. Tumor volume was calculated using the ellipsoid formula: length× width× height×0.523.

Maleimide-DyLight 680 conjugation – 15–fold molar excess of Tris(2-carboxyethyl)phosphine (TECP, Sigma-Aldrich, C4706) were used to reduce IgG1s for 2 hours at room temperature. A 10-fold molar excess of DyLight 680 (ThermoFisher Scientific, Cat. no. 46618) was then added and the reaction mixture was kept at room temperature to react for 2 hours. Pierce Dye Removal Column kit (ThermoFisher Scientific, Cat. no. 22858) was used to remove excess dye and conjugation efficiency was determined using Nanodrop ND-1000 spectrophotometer to calculate the molar ratio of DyLight 680 to protein.

Bio-distribution determination – Bio-distribution of DyLight 680-labeled anti-MSLN IgG1s in N87/Capan-2 tumor-bearing mice at 24 hours post-injection (0.5 nmol, 150 µL per injection) were determined *ex vivo* with NIRF (near infrared fluorescence) using a small-animal IVIS imaging system (IVIS-Spectrum, Xenogen) with excitation and emission wavelengths of 675 and 720 nm. Fluorescence emission was normalized to photons per second per centimeter squared per steradian (p/s/cm2/sr). The mean values and standard deviations are calculated with three independent measurements.

Ex vivo NIRF imaging – Tumor bearing mice were intravenously injected with 0.5 nmol of DyLight 680-anti-MSLN IgG1 antibodies and isotype control IgG1 (150 µL per injection; 3 mg/kg). Mice were euthanized after 24 hours post-injection. The tumor, organs and blood were collected and NIRF imaged using a small-animal IVIS imaging system (IVIS-Spectrum, Xenogen) with excitation 675 nm and emission 720 nm wavelengths. Fluorescence emission was normalized to photons per second per centimeter squared per steradian (p/s/cm2/sr).

Serum biochemical analysis – 10-week-old male NOD/SCID mice (BioLASCO Taiwan Co. Ltd) were intraperitoneally injected with 15 mg/kg anti-MSLN IgG1-vcMMAEs once a week for a total

of three doses. The blood samples were collected three weeks after the last does and assayed for alanine transaminase (ALT), alkaline phosphatase (ALP), creatinine (CRE) and blood urea nitrogen (BUN) using Fuji Dri-Chem 4000i (Fujifilm Europe) according to manufacturer's instructions.

II. Supplementary Figures

Supplementary Figure S1. Binding of the scFv CDR-variants of M9 to MSLN expressed on the cell surfaces of two types of cultured human cancer cells. Mean fluorescence intensities (MFIs) for each of the AL1-RFP-conjugated scFv CDR-variants of M9 binding to N87 and H226 cells are shown in the y-axis and x-axis respectively. (A) The raw output MFI readings were recorded with BD FACS Canto II Cell Analyser. (B) The raw output MFI readings were recorded with BD LSR Fortessa Flow Cytometer. All the MFI readings were determined with 10000 events from each well containing $1\sim5x10^5$ cells/well cells. These two panels were measurements from two groups of non-overlapping scFv CDR-variants of M9, and are used to show that the expression level of MSLN on H226 is higher than that on N87. The data points for the M9 scFv are colored in red; the data points in grey and blue are the scFv CDR-variants of M9, for which the sequences are shown in Supplementary Table S1. The blue data points are the scFv candidates selected, in the studies followed, to be reformatted into human IgG1 framework for further characterizations as ADC candidates against MSLN. Numerical data and CDR sequence for each of the data points are shown in Supplementary Table S1.

(A)

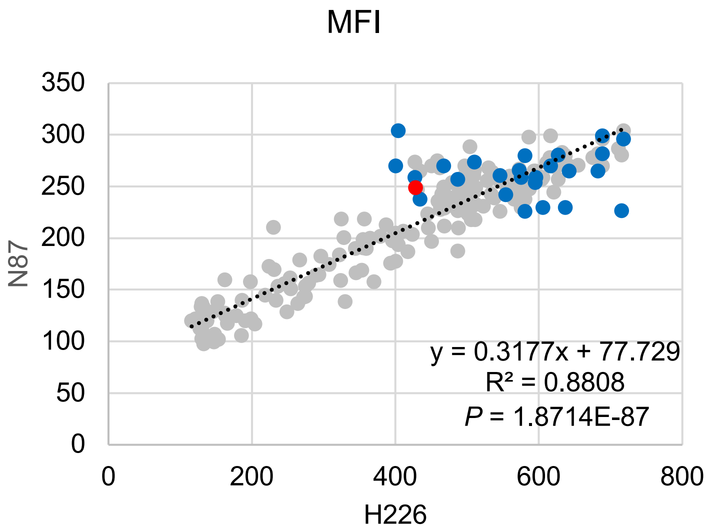

MFI
350
300
250
200
150
100
50
0
N87
0
200
400
600
800
H226
y = 0.3177x + 77.729
R² = 0.8808
P = 1.8714E-87

(B)

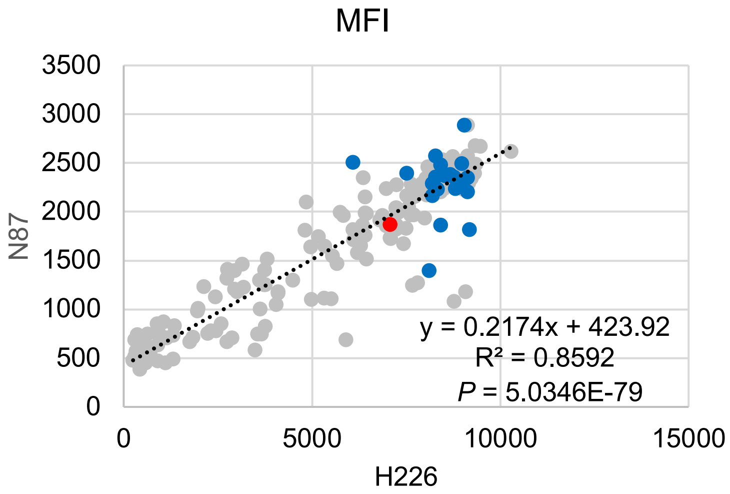

MFI
3500
3000
2500
2000
1500
1000
500
0
N87
0
5000
10000
15000
H226
y = 0.2174x + 423.92
R² = 0.8592
P = 5.0346E-79

Supplementary Figure S2. HIC characterization of IgG1-vc-MMAEs. Hydrophobic interaction chromatography (HIC) analyses of the IgG1s and their corresponding IgG1-vc-MMAEs on a butyl-NPR column yielded three predominant peaks (labelled in red numbers) corresponding to IgG1s containing zero, two and four drug molecules per IgG1 respectively. The peaks for IgG1-M9 and M9-vcMMAE did not appear in the range of the HIC elution condition.

<table>
<tr><td colspan="2" rowspan="2">HIC elution program and buffers</td><td colspan="2">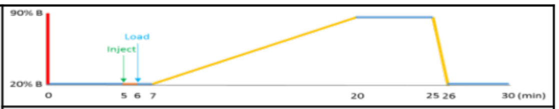
</td></tr>
<tr><td colspan="2">Buffer A: potassium phosphate buffer, pH 7.2, 50 mM; 1.5 M Ammonium Sulfate.
Buffer B: potassium phosphate buffer, pH 7.2, 50 mM</td></tr>
<tr><td>Name</td><td>Format</td><td>HIC analysis</td><td>DAR</td></tr>
<tr><td rowspan="2">SS1</td><td>IgG1</td><td>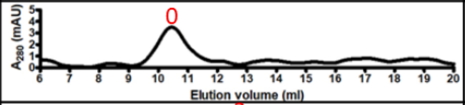
</td><td>0</td></tr>
<tr><td>IgG1-vcMMAE</td><td>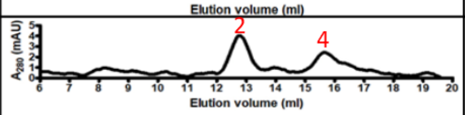
</td><td>2.58</td></tr>
<tr><td rowspan="2">M9</td><td>IgG1</td><td>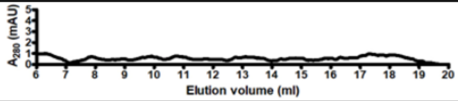
</td><td>0</td></tr>
<tr><td>IgG1-vcMMAE</td><td>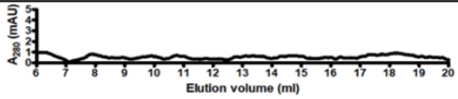
</td><td>ND</td></tr>
<tr><td rowspan="2">HS2X-02
(CHS5)</td><td>IgG1</td><td>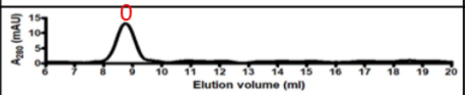
</td><td>0</td></tr>
<tr><td>IgG1-vcMMAE</td><td>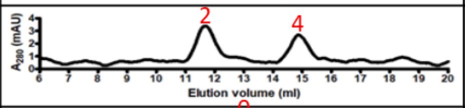
</td><td>2.56</td></tr>
<tr><td rowspan="2">HSA1_19
(CHS7)</td><td>IgG1</td><td>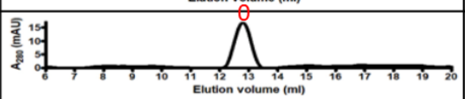
</td><td>0</td></tr>
<tr><td>IgG1-vcMMAE</td><td>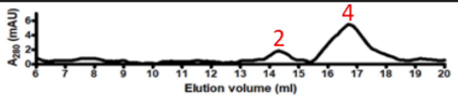
</td><td>3.23</td></tr>
<tr><td rowspan="2">HSX1_34
(CHS8)</td><td>IgG1</td><td>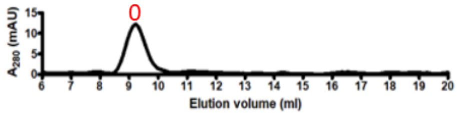
</td><td>0</td></tr>
<tr><td>IgG1-vcMMAE</td><td>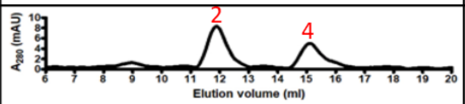
</td><td>2.63</td></tr>
<tr><td rowspan="2">ALA12</td><td>IgG1</td><td>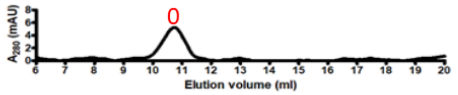
</td><td>0</td></tr>
<tr><td>IgG1-vcMMAE</td><td>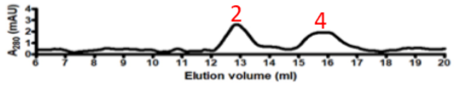
</td><td>2.72</td></tr>
</table>

Supplementary Figure S3. DAR plotted against IC_{50} for the anti-MSLN IgG1-vcMMAEs. The numerical data for the ADCs are listed in Supplementary Table S1. The data points with the y-axis value assigned as ND (Not Determined) are not used for correlation calculations.

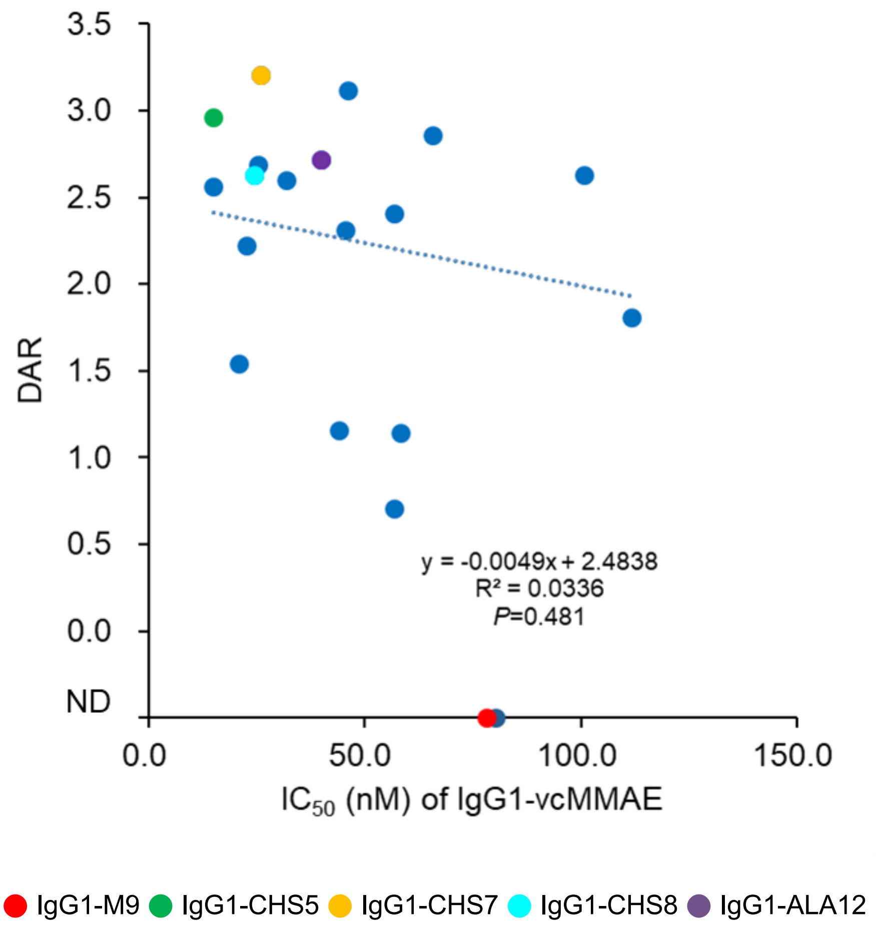

Supplementary Figure S4. The scFv VL/VH variable domain structures computationally modelled using RosettaAntibody with default parameters. Amino acid residues in van der Waals spheres are labeled according to the Kabat numbering as shown in the M9 scFv structure. The secondary structures of the scFvs are shown by the arrow ribbons connected with cylindrical loops.

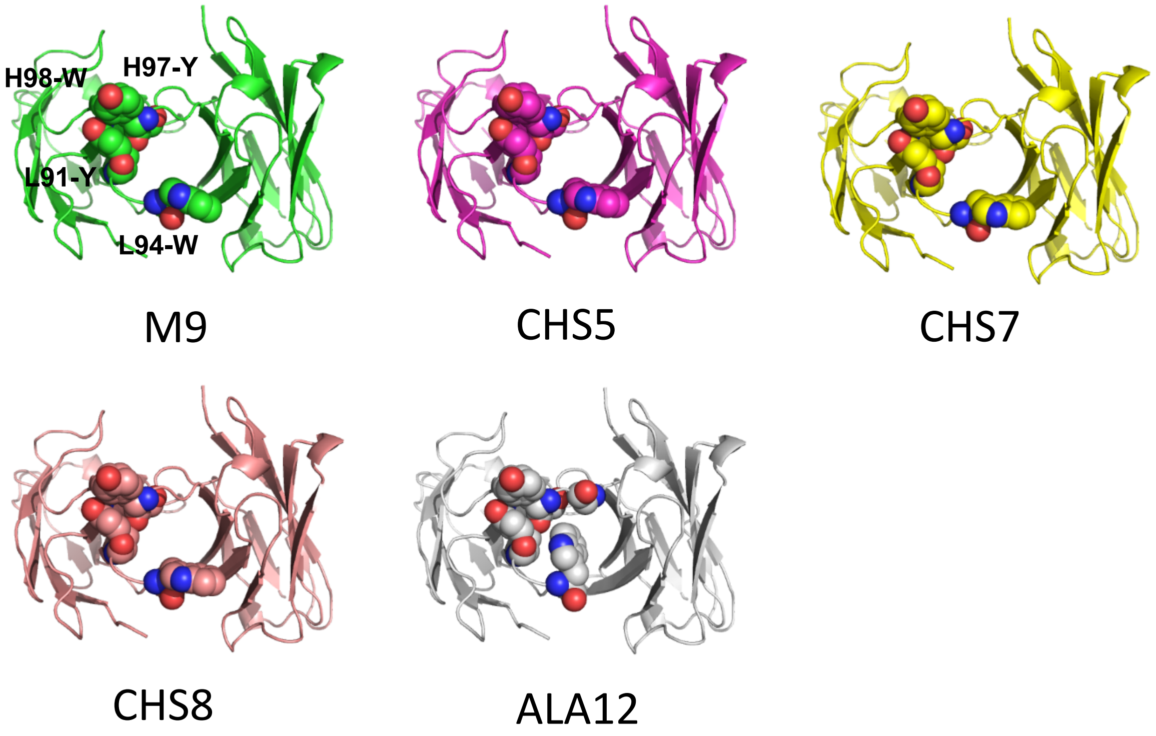

Supplementary Figure S5. Full-length Western blots for actin and MSLN. The full-length blots without brightness and contract adjustment in panels (A) to (D) are presented in Figure 6A. Different adjustments of brightness and contract for each blot image are shown in this figure for comparisons. Images of the actin (as internal control) and MSLN signals in the full-length blots were taken according to the experimental procedure described in Supplementary Methods.

(A)

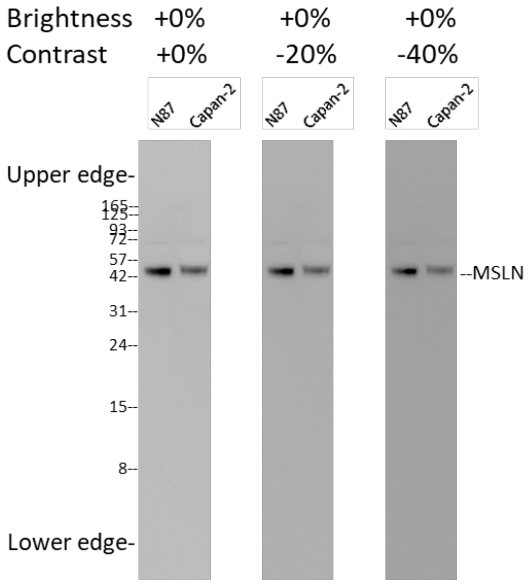

(B)

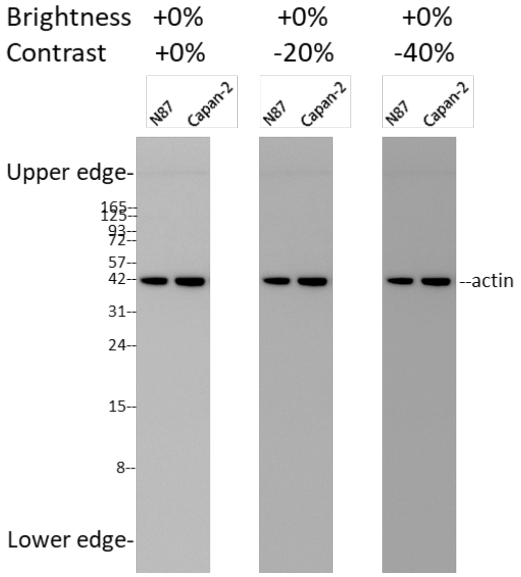

(C)

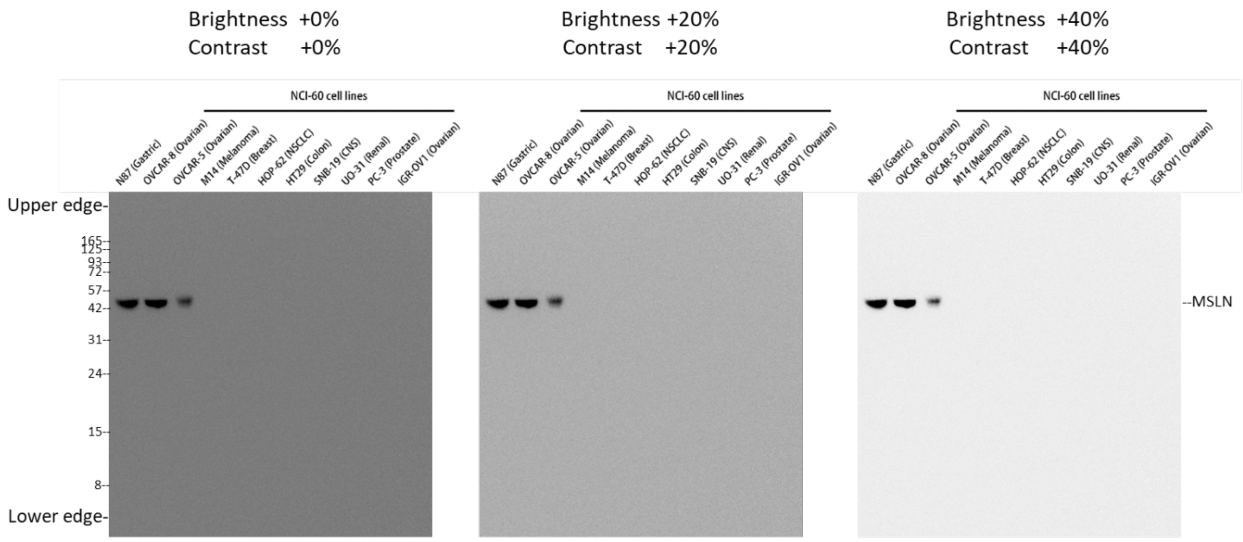
Brightness +0%
Contrast +0%
Brightness +20%
Contrast +20%
Brightness +40%
Contrast +40%
NCI-60 cell lines
N87 (Gastric)
OVCAR-8 (Ovarian)
OVCAR-5 (Ovarian)
M14 (Melanoma)
T-47D (Breast)
HOP-62 (NSCLC)
HT29 (Colon)
SNB-19 (CNS)
UO-31 (Renal)
PC-3 (Prostate)
IGR-OV1 (Ovarian)
Upper edge-
165
125
93
72
57
42
31
24
15
8
Lower edge-
--MSLN

(D)

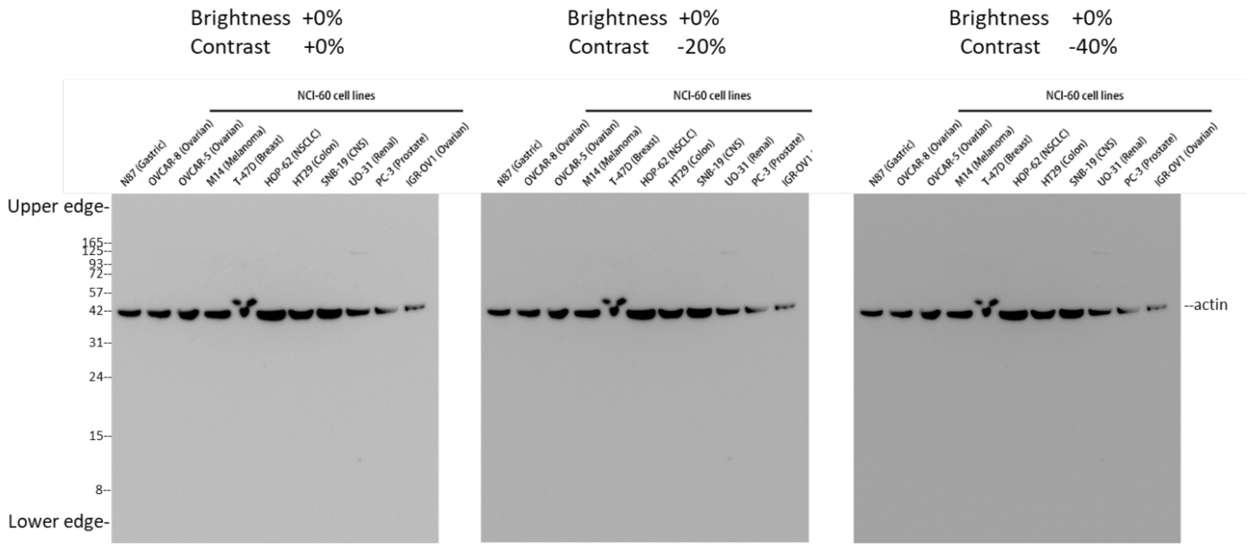
Brightness +0%
Contrast +0%
Brightness +0%
Contrast -20%
Brightness +0%
Contrast -40%
NCI-60 cell lines
N87 (Gastric)
OVCAR-8 (Ovarian)
OVCAR-5 (Ovarian)
M14 (Melanoma)
T-47D (Breast)
HOP-62 (NSCLC)
HT29 (Colon)
SNB-19 (CNS)
UO-31 (Renal)
PC-3 (Prostate)
IGR-OV1 (Ovarian)
Upper edge-
165
125
93
72
57
42
31
24
15
8
Lower edge-
--actin

Supplementary Figure S6. Ex vivo NIRF imaging of excised organs of N87/Capan-2 tumor bearing mice. Images are shown for the excised tumors and organs of (A) N87 tumor-bearing mice and (B) Capan-2 tumor-bearing mice. The mice were euthanized at 24 hours after intravenous injection of DyLight 680-labeled anti-MSLN IgG1s. Experimental details are described in Supplementary Methods.

(A)

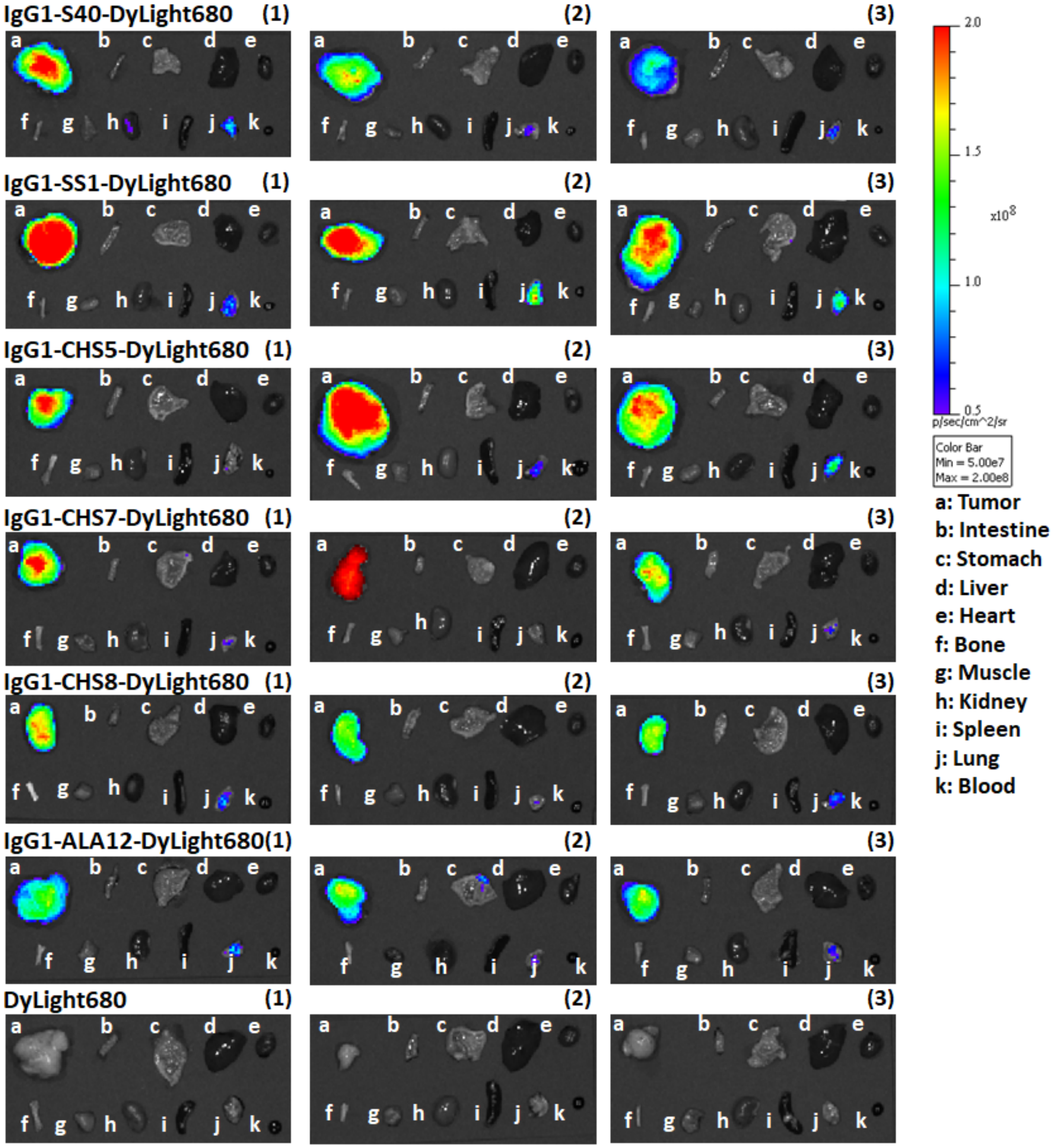

(B)

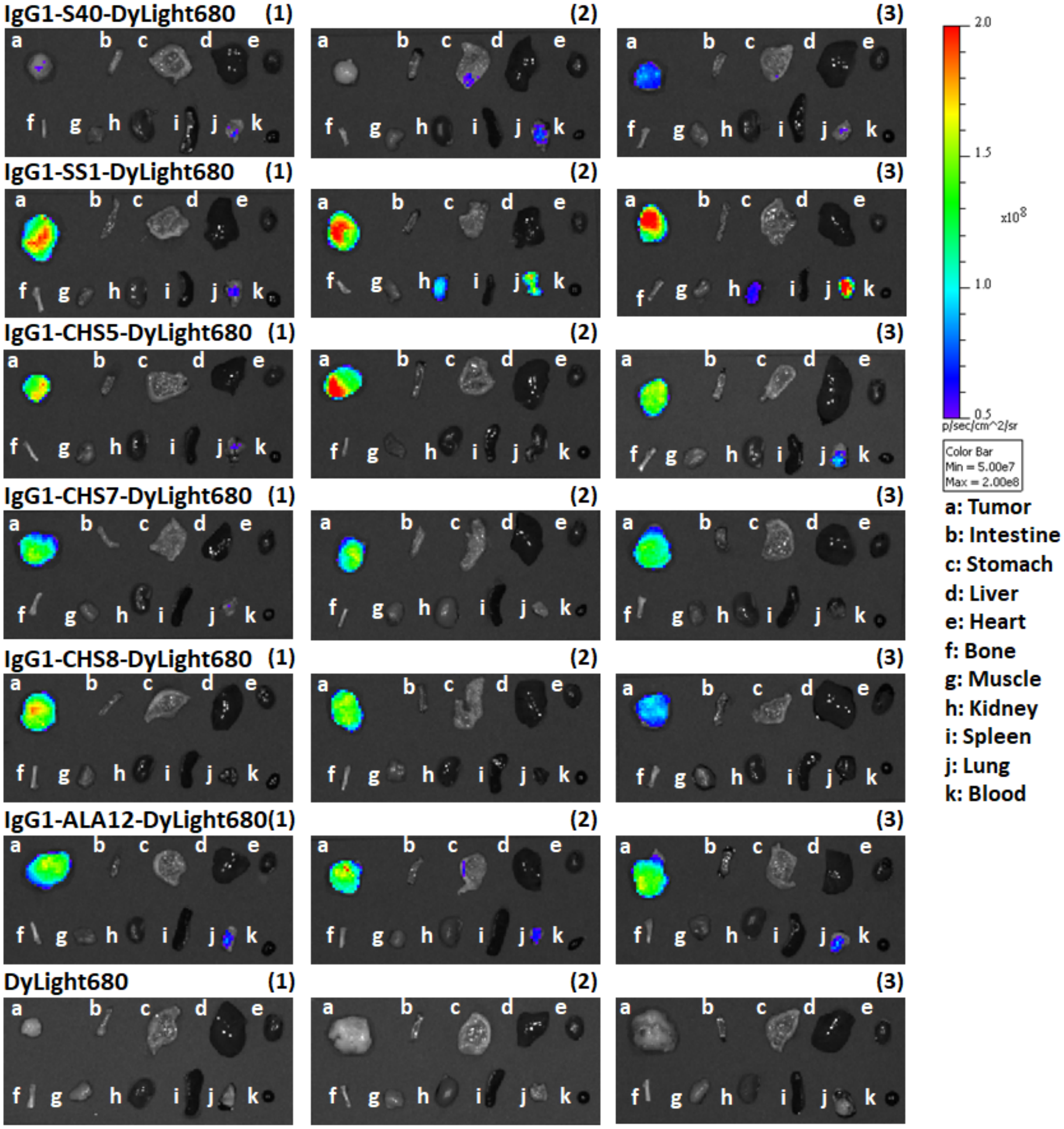

IgG1-S40-DyLight680
IgG1-SS1-DyLight680
IgG1-CHS5-DyLight680
IgG1-CHS7-DyLight680
IgG1-CHS8-DyLight680
IgG1-ALA12-DyLight680
DyLight680
(1)
(2)
(3)
a b c d e f g h i j k
2.0
1.5
x10^8
1.0
0.5
p/sec/cm^2/sr
Color Bar
Min = 5.00e7
Max = 2.00e8
a: Tumor
b: Intestine
c: Stomach
d: Liver
e: Heart
f: Bone
g: Muscle
h: Kidney
i: Spleen
j: Lung
k: Blood

III. Supplementary Tables

Supplementary Table S1. scFv CDR-variants of M9 derived from Step 5~6 of the procedure shown in Figure 1. The antibodies, for which the CDR sequences and numerical data colored in blue, green (CHS5), orange (CHS7), cyan (CHS8), and purple (ALA12), were reformatted with human IgG1 framework for downstream characterization as antibody candidates for ADCs against MSLN. The numerical data and CDR sequences for M9 are colored in red. The binding and cytotoxicity characterizations of these scFv CDR-variants of M9 are shown in Figures 3~5. Blanks indicate data that have not been measured; ND indicates the measurement has been carried out but the data was not sufficient to determine the DAR. Conserved aromatic residues in M9, CHS5,7,8 and ALA12 shown in Supplementary Figure S4 are underlined in the CDRs.

ID	Viability H226 (%)	Viability N87 (%)	CamSol score	RH-score	MFI(%) H226	MFI(%) N87	L1	L2	L3	H1	H2	H3	ADC yield (%)	HIC retention time(min.)	DAR	IgG1 EC_{50}(ng/mL)	IgG1-vcMMAE EC_{50} (ng/mL)	IgG1-AL1-PE38KDEL IC_{50}(nM)	IgG1-vcMMAE IC_{50}(nM)
M9	56.7	80.6	-0.16	0	69	64	RASQDVNDGVA	SGSPWLYS	QQYFNWPIT	AASGFTIDNYGIH	WIWPYGGSTY	ARGYYWYDY	17.5%	18.0	ND	3.15	4.86	0.391	78.13
HS2X-02(CHS5)	21.0	46.1	0.92	13	71	78	RASQDVKEGVA	DQSHRLYS	QQYYAWPST	AASGFTITDRTIH	SIFPTKGVTT	ARGRYWMDY	109.3%	3.8	2.56	4.67	9.56	0.056	15.03
HSA1_19(CHS7)	8.7	48.3	1.08	11	90	87	RASQDVEDGVA	GIKDLLYS	QQYYRWPST	AASGFTIDKEAIH	SIYPHSGFTL	ARGRYWMDY	93.2%	7.7	3.21	10.3	16.27	0.056	25.95
HSX1_34(CHS8)	72.5	76.1	1.18	14	31	32	RASQDVKEGVA	DERERLYS	QQYNTWPAT	AASGFTISKASIH	SIWPTKGFTT	ARGRYWLDY	101.8%	4.2	2.63	5.05	9.26	0.0084	24.57
ALA12	9.1	44.8	0.07	5	89	100	RASQDVRDGVA	DQMVRLYS	QQYFNWPVT	AASGFTIGNWTIH	LIWPESGATL	ARGRYWMDY	93.9%	5.7	2.72	8.37	10.49	0.10	39.94
HJ3_04C	9.0	48.3	0.72	9	96	100	RASQDVEEAVA	SGRETLYS	QQYFRWPVT	AASGFTIDNYGIH	SIFPRSGFTL	ARGRYWYDY	59.0%	6.6				0.108	
HJ6_04H	8.0	38.9	0.76	9	97	99	RASQDVNDGVA	GRNDILYS	QQYFRWPVT	AASGFTIDTRPIH	SIFPRSGFTL	ARGRYWYDY	74.5%	10.4		6.75	7.81	0.209	
WY1X-01A	4.2	35.9	0.79	8	85	81	RASQDVTDGVA	STGNRLYS	QQIYRWPST	AASGFTIGKTGIH	GIYPQVGVTM	ARGRYFYDY	86.6%					0.079	
HJ6_10C	8.6	52.9	0.34	7	95	96	RASQDVNDGVA	DENVRLYS	QQYFNWPIT	AASGFTIDTRPIH	WIWPYGGSTY	ARGRYWYDY	70.1%	5.8		7.92	8.22	0.104	
HS1A-02	4.4	37.2	0.39	7	88	86	RASQDVNDGVA	THTIGLYS	QQYFRWPST	AASGFTISTLPIH	TIHPSRGITL	ARARYWFDY	92.8%	4.7		5.93	7.53	0.084	
HJ7_05E	15.2	38.1	0.25	6	91	100	RASQDVEEAVA	SGRETLYS	QQYFRWPVT	AASGFTIDTRPIH	WIWPYGGSTY	ARGYYWYDY	52.6%			6.49	7.13	0.071	
HS1A-05	9.3	51.7	0.60	4	93	87	RASQDVRKGVA	GVNNHLYS	QQYYRWPAT	AASGFTISANPIH	WIWPYGGSTY	ARGRYWLDY	76.6%			5.50	7.26	0.051	
YC1-03C	5.2	32.2	0.41	4	82	82	RASQDVGGGVA	THPIGLYS	QQYNTWPVT	AASGFTIMTKSIH	SIYPRAGVTM	ARGRYFYDY	80.0%	8.2	2.20	2.87	2.90	0.068	

CY1-B10	9.1	41.6	0.08	3	71	70	RASQDVGGGVA	GALGRLYS	QQYFNWPTT	AASGFTIKTFAIH	SIWPQVGVTT	ARGRFWMDY	64.0%	9.3	0.47	5.51	6.82	0.114	
WY2A-07H	13.8	46.7	-0.14	1	80	78	RASQDVLGGVA	GYNQLLYS	QQYITWPVT	AASGFTIMSMPIH	TIYPHRGFTT	ARGRYWMDY	24.7%					0.168	
YC1-02G	14.1	41.7	-0.09	1	81	79	RASQDVPEGVA	DRERVLYS	QQYFNWPVT	AASGFTILPGPIH	WIWPYGGSTY	ARGYYWLDY	68.1%	14.9	ND	2.83	2.91	0.123	
HS1A-15	7.7	43.4	0.14	0	99	98	RASQDVMDGVA	GWNNVLYS	QQYFNWPIT	AASGFTIDQNPIH	GIYPSGGITL	ARGLYWLDY	78.4%					0.119	
CPT_034	6.8	43.7	0.09	2	94	89	RASQDVNDGVA	SGSPWLYS	QQYFRWPVT	AASGFTIDTRPIH	WIWPYGGSTY	ARGYYWYDY	5.3%					0.281	156.10
YC1-08E	99.3	100.0	0.55	11	53	36	RASQDVRDGVA	GFHHLLYS	QQYNNWPVT	AASGFTIERSRIH	RIHPLSGATT	ARGFYWLDY	76.1%	6.1	1.81	6.33	7.56	0.554	111.80
HS2A-05	11.0	41.0	0.26	2	72	93	RASQDVPEGVA	GWNNHLYS	QQYFIWPVT	AASGFTIPRAGIH	AIMPGKGITL	ARGRYWYDY	70.6%	5.5	2.63	6.49		0.153	100.60
HS2X14	5.4	35.2	0.13	5	89	81	RASQDVNDGVA	GWNNMLYS	QQYFNWPVT	AASGFTINSYSIH	SIHPSHGITM	ARGRYWLDY	62.0%	8.2		8.10	11.51	0.114	80.83
PHT2-D08	7.4	36.4	-0.18	1	87	85	RASQDVGVGVA	GVAGLLYS	QQYNTWPVT	AASGFTITTAGIH	SIHPSIGFTL	ARGRYWLDY	43.0%	14.5	ND	2.73	2.21	0.070	80.24
CPT_122	3.9	30.0	0.91	11	99	88	RASQDVEEAVA	DENVRLYS	QQYFRWPVT	AASGFTIDTRPIH	SIWPSRGYTT	ARGRYWYDY	87.9%	5.0				0.107	80.04
CY1-D01	6.6	61.2	0.79	8	91	87	RASQDVQDGVA	DESKRLYS	QQYFNWPST	AASGFTIYPSPIH	SIYPMRGTTL	ARGRFWLDY	53.0%			6.33	7.77	0.095	73.40
SCC1R-28	10.9	44.1	0.17	8	82	76	RASQDVGGGVA	DRVTVLYS	QQYHNWPIT	AASGFTISERTIH	LIWPFSGTTI	ARGRYWTDY	75.0%	6.9	2.86			0.094	65.63
YCM4-05G	15.3	49.9	0.50	8	100	96	RASQDVNDGVA	GRNDILYS	QQYFRWPVT	AASGFTIRMSTIH	SIFPRSGFTL	ARGRYWYDY	48.0%					0.204	58.37
CY1-E12	9.5	56.4	0.90	8	100	93	RASQDVNDGVA	GHDGLLYS	QQYYRWPTT	AASGFTITARTIH	SIYPYRGMTL	ARGRYWLDY	39.0%	6.1	1.14	8.82	8.47	0.493	58.26
CY1-G02	11.1	61.9	0.41	6	88	76	RASQDVANGVA	DRMEILYS	QQYSTWPVT	AASGFTIAQQTIH	SIGPERGWTM	ARARYWFDY	73.0%	5.3	0.71	3.88	4.99	0.153	56.71
SCC2R-24	22.4	49.4	0.68	10	80	81	RASQDVGDGVA	DQMERLYS	QQYFNWPVT	AASGFTINHSAIH	DIRPSSGITL	ARGRYWIDY	65.0%	4.8	2.41			0.121	56.69
HJ4_03G	6.1	49.6	0.55	7	76	81	RASQDVEEAVA	GRNDILYS	QQYFRWPVT	AASGFTIDNYGIH	SIFPRSGFTL	ARGTYWYDY	58.0%	7.3				0.130	55.60
YK-69	13.5	51.7	0.91	11	51	85	RASQDVNDGVA	DENVRLYS	QQYFRWPVT	AASGFTIDTRPIH	SIWPSRGYTT	ARGRYYYDY	51.9%			7.13	7.70	0.616	54.11
YK-38	11.0	38.7	0.20	7	88	89	RASQDVSRGVA	DENVRLYS	QQYFRWPVT	AASGFTIRMSTIH	WIWPYGGSTY	ARGRYWYDY	47.6%			8.08	8.16	0.086	47.26
HS1X-02	20.2	62.0	0.94	10	82	69	RASQDVGDGVA	SRFDLLYS	QQYSRWPTT	AASGFTIHRETIH	SIFPYRGFTL	ARGGYWLDY	62.5%			6.71		0.094	46.52
SCC1G-56	47.3	61.8	1.01	12	59	62	RASQDVTDGVA	DRSTWLYS	QQYSRWPTT	AASGFTIGRHSIH	SIFPSRGATL	ARGRFYYDY	75.0%	5.6	3.12			0.050	46.00
PHT2-A12	4.7	31.4	0.85	9	84	86	RASQDVKDGVA	GIKDLLYS	QQYFRWPST	AASGFTIGTTPIH	SIFPGKGFTT	ARGRYWIDY	66.0%	7.4	2.31	7.08	10.11	0.070	45.59
PHT2-E02	15.0	46.7	-0.33	0	73	74	RASQDVAEAVA	DQTIMLYS	QQYFIWPVT	AASGFTIYGSPIH	LIYPDRGLTV	ARGRFWLDY	69.0%	8.5	1.16	7.70	8.37	0.068	44.12
HJ4_04H	8.1	51.2	0.47	8	100	100	RASQDVNDGVA	SGRETLYS	QQYFRWPVT	AASGFTIDTRPIH	WIWPYGGSTY	ARGRYWYDY	66.0%	5.9				0.186	43.18
YCM4-10G	100.0	99.6	0.43	8	31	54	RASQDVSGGVA	GRNDILYS	QQYFRWPVT	AASGFTISHSTIH	SIFPRSGFTL	ARGRYYYDY	46.0%	6.6				0.230	39.30
HJ5_11B	7.6	33.7	0.64	9	89	94	RASQDVNDGVA	DENVRLYS	QQYFRWPVT	AASGFTIDTRPIH	SIWPSRGYTT	ARGYYWYDY	73.7%	7.0		6.42	7.01	0.387	36.11

HS1X-32	11.7	48.0	0.88	9	88	88	RASQDVSDGVA	GWDNRLYS	QQYYRWPST	AASGFTINRNAIH	SIWPHLGITT	ARGRYWYDY	27.4%			7.25	8.44	0.171	35.41
YK-85	13.5	51.6	0.87	10	83	97	RASQDVNDGVA	GRNDILYS	QQYFRWPVT	AASGFTIDTRPIH	SIWPSRGYTT	ARGRYYYDY	81.6%			5.51	7.85	0.078	32.67
SCC2G-33	12.7	38.5	0.96	13	89	89	RASQDVEDGVA	DRRRVLYS	QQYFRWPTT	AASGFTITRGVIH	NITPSRGYTM	ARGRYWLDY	70.0%	4.3	2.60			0.107	31.93
HJ5_05H	8.0	46.5	0.71	9	94	94	RASQDVNDGVA	GRNDILYS	QQYFNWPIT	AASGFTIDTRPIH	SIWPSRGYTT	ARGRYWYDY	64.0%	5.4				0.201	30.65
HS1X-18	38.1	53.7	0.35	12	87	71	RASQDVGKGVA	DQSRRLYS	QQYFNWPVT	AASGFTISRVTIH	SIYPTRGHTS	ARGRYWIDY	86.8%	3.6		6.21	8.33	0.621	28.21
HS1X-09	8.1	39.4	0.79	7	67	71	RASQDVEEGVA	NGPIGLYS	QQYHNWPTT	AASGFTILKSAIH	VIFPYKGMTM	ARGRYWKDY	79.1%					0.333	27.49
HS1A-06	5.5	48.6	0.74	8	91	90	RASQDVKEGVA	SGPLGLYS	QQYNTWPVT	AASGFTISGHTIH	SINPSRGFTM	ARGRYWLDY	99.9%	4.5		6.28	6.32	0.077	26.05
YC1-01C	4.7	36.5	0.68	9	79	81	RASQDVSGGVA	DRVEILYS	QQHFNWPVT	AASGFTIGSRSIH	SIYPYKGLTS	ARGRFWLDY	82.1%	5.0	2.69	3.98	5.36	0.101	25.45
HJ4_06A	9.9	44.5	0.68	9	95	83	RASQDVSRGVA	DENVRLYS	QQYFRWPVT	AASGFTIDNYGIH	SIFPRSGFTL	ARGRYWYDY	60.0%	8.3				0.151	24.16
PHT2-G02	74.9	61.1	0.31	-4	61	63	RASQDVTDGVA	SQMALLYS	QQYNSWPPT	AASGFTIPMVPIH	LIWPPNGLTL	ARGQYWLDY	70.0%	10.5	2.22	8.17	9.85	0.094	22.66
HS1X-17	15.2	48.6	0.68	11	83	84	RASQDVQDGVA	DRDKVLYS	QQYFNWPTT	AASGFTIRKTPIH	SISPLKGFTF	ARGRFWLDY	85.8%					0.131	22.09
HJ4_07G	7.4	36.9	0.45	7	85	86	RASQDVNDGVA	DENVRLYS	QQYFRWPVT	AASGFTIDNYGIH	SIFPRSGFTL	ARGYYWYDY	27.0%	12.6				0.139	21.55
HJ5_04F	4.4	36.2	0.57	8	65	81	RASQDVNDGVA	DENVRLYS	QQYFRWPVT	AASGFTIDTWPIH	SIFPRSGFTL	ARGRYWYDY	63.0%	5.9				0.117	21.51
CY1-C06	99.0	110.6	0.75	10	54	43	RASQDVEEGVA	AQTRTLYS	QQYHTWPTT	AASGFTIGGSTIH	LILPKQGNTV	ARGRFWLDY	82.0%	3.7	1.54	8.82	6.65	0.185	20.81
HJ1_03G	103.1	105.4	0.75	9	47	44	RASQDVSRGVA	GRNDILYS	QQYFRWPVT	AASGFTIDTRPIH	SIFPRSGFTL	ARGRYWYDY	33.0%	6.3				0.102	20.59
HJ7_01F	14.9	39.3	0.51	8	89	89	RASQDVNDGVA	SGRETLYS	QQYFRWPVT	AASGFTIDNYGIH	SIWPSRGYTT	ARGRYYYDY	95.3%	3.6		6.44	7.90	0.133	10.58
YK-88	39.6	55.5	0.79	10	52	80	RASQDVNDGVA	SGRETLYS	QQYFRWPVT	AASGFTIDNYGIH	SIWPSRGYTT	ARGRYYYDY	89.0%			6.17	8.55	0.130	8.86
HJ1_06F	67.4	94.5	0.20	5	89	83	RASQDVSGGVA	GRNDILYS	QQYFNWPIT	AASGFTIDTRPIH	WIWPYGGSTY	ARGRYWYDY							
HJ1_07G	106.0	120.2	0.36	7	8	6	RASQDVNDGVA	SDKRGLYS	QQYFNWPIT	AASGFTIDNYGIH	SIFPRSGFTL	ARGYYWYDY							
HJ1_09C	101.9	123.5	0.23	4	26	19	RASQDVNDGVA	SGSPWLYS	QQYFRWPVT	AASGFTIRMSTIH	SIWPSRGYTT	ARGYYWYDY							
HJ1_10D	29.1	103.9	0.31	6	57	61	RASQDVNDGVA	DENVRLYS	QQYFNWPIT	AASGFTIDNYGIH	SIFPRSGFTL	ARGYYWYDY							
HJ1_10F	99.9	119.9	0.56	4	18	32	RASQDVSGGVA	SGSPWLYS	QQYFNWPIT	AASGFTIDTRPIH	SIFPRSGFTL	ARGSIGMDY							
HJ2_04A	99.1	111.0	0.58	8	16	19	RASQDVSRGVA	GRNDILYS	QQYFRWPVT	AASGFTIDTRPIH	SIWPSRGYTT	ARGYYWYDY							
HJ2_04C	84.4	82.5	0.31	3	73	78	RASQDVNDGVA	SGSPWLYS	QQYFNWPIT	AASGFTIDNYGIH	SIWPSRGYTT	ARGVLLVDY							
HJ2_04E	92.9	92.8	0.12	2	58	53	RASQDVSGGVA	SGSPWLYS	QQYFRWPVT	AASGFTIDNYGIH	WIWPYGGSTY	ARGRYWYDY							
HJ2_05B	102.1	92.3	0.23	3	31	35	RASQDVSRGVA	SGSPWLYS	QQYFNWPIT	AASGFTIDNYGIH	SIWPSRGYTT	ARGYYWYDY							
HJ2_05F	85.2	79.8	0.14	5	46	46	RASQDVEEAVA	GRNDILYS	QQYFNWPIT	AASGFTIRMSTIH	SIFPRSGFTL	ARGYYWYDY							

HJ2_06F	79.6	88.5	0.09	3	25	18	RASQDVNDGVA	SGSPWLYS	QQYFNWPIT	AASGFTIRMSTIH	SIWPSRGYTT	ARGYYWYDY							
HJ2_11B	75.6	87.6	0.52	5	34	39	RASQDVNDGVA	SGSPWLYS	QQYFNWPIT	AASGFTIDNYGIH	SIWPSRGYTT	ARGRYYYDY							
HJ3_04F	5.2	43.1	0.54	8	60	75	RASQDVSGGVA	DENVRLYS	QQYFNWPIT	AASGFTIDNYGIH	SIWPSRGYTT	ARGRYWYDY							
HJ4_06G	37.7	59.2	0.08	4	93	87	RASQDVNDGVA	GRNDILYS	QQYFRWPVT	AASGFTIDNYGIH	WIWPYGGSTY	ARGYYWYDY							
HJ4_08B	15.0	49.3	0.23	6	84	70	RASQDVNDGVA	DENVRLYS	QQYFRWPVT	AASGFTIDTRPIH	WIWPYGGSTY	ARGYYWYDY							
HJ4_10E	6.3	38.5	0.40	6	62	77	RASQDVNDGVA	GRNDILYS	QQYFRWPVT	AASGFTIDNYGIH	SIFPRSGFTL	ARGYYWYDY							
HJ6_01B	8.7	46.2	0.51	8	64	75	RASQDVSRGVA	DENVRLYS	QQYFRWPVT	AASGFTIDNYGIH	SIWPSRGYTT	ARGYYWYDY							
HJ6_11E	26.4	67.9	0.50	8	99	91	RASQDVNDGVA	GRNDILYS	QQYFRWPVT	AASGFTIRMSTIH	SIFPRSGFTL	ARGRYWYDY							
HJ6_12A	108.1	111.8	0.50	9	2	8	RASQDVSRGVA	DENVRLYS	QQYFNWPIT	AASGFTIRMSTIH	SIWPSRGYTT	ARGRYYYDY							
HJ6_12D	6.7	42.8	0.19	6	73	73	RASQDVSGGVA	DENVRLYS	QQYFRWPVT	AASGFTIRMSTIH	SIFPRSGFTL	ARGYYWYDY							
HJ7_03A	81.6	90.5	0.85	12	12	7	RASQDVGDTVA	SDKRGLYS	QQYFRWPVT	AASGFTIDTRPIH	SIWPSRGYTT	ARGRYWYDY							
HJ7_03H	90.0	90.5	0.85	11	2	4	RASQDVNDGVA	SDKRGLYS	QQYFRWPVT	AASGFTIDNYGIH	SIWPSRGYTT	ARGRYYYDY							
HJ7_04B	89.1	82.7	0.59	9	5	0	RASQDVSGGVA	SDKRGLYS	QQYFRWPVT	AASGFTIDTRPIH	SIWPSRGYTT	ARGYYWYDY							
HJ7_04H	35.6	66.7	0.06	4	70	67	RASQDVGDSVA	SGKVTLYS	QQYFRWPVT	AASGFTIRMSTIH	WIWPYGGSTY	ARGGYCYDY							
HJ7_05B	94.4	91.0	0.80	7	35	48	RASQDVEEAVA	SGSPWLYS	QQYFRWPVT	AASGFTIDTRPIH	SIWPSRGYTT	ARGRYYYDY							
HJ7_06E	104.2	106.3	0.51	4	13	26	RASQDVSGGVA	SGSPWLYS	QQYFRWPVT	AASGFTIDTRPIH	SIFPGSGFTL	ARGRYYYDY							
HJ7_06F	32.1	74.9	0.33	7	55	46	RASQDVNDGVA	DENVRLYS	QQYFRWPVT	AASGFTIRMSTIH	SIFPLSGFTL	ARGRYYYDY							
HJ7_08F	39.7	78.6	0.52	9	75	76	RASQDVNDGVA	SDKRGLYS	QQYFRWPVT	AASGFTIDTRPIH	WIWPYGGSTY	ARGKYWYDY							
HJ7_09B	11.7	57.2	0.67	6	71	75	RASQDVNDGVA	SGSPWLYS	QQYFRWPVT	AASGFTIDTRPIH	SIFPRSGFTL	ARGRYWYDY							
HJ7_09F	87.2	86.3	0.45	8	39	47	RASQDVSGGVA	SDKRGLYS	QQYFRWPVT	AASGFTIDTRPIH	WIWPYGGSTY	ARGRYYYDY							
HJ7_10G	86.9	86.1	0.66	6	38	30	RASQDVNDGVA	SGSPWLYS	QQYFRWPVT	AASGFTIDNYGIH	SIWPSRGYTT	ARGRYYYDY							
HJ7_11A	98.8	91.1	0.43	8	2	0	RASQDVNDGVA	DENVRLYS	QQYFNWPIT	AASGFTIRMSTIH	SIFPRSGFTL	ARGRYYYDY							
HJ7_12C	21.0	79.6	0.02	4	84	82	RASQDVSGGVA	DENVRLYS	QQYFRWPVT	AASGFTIDNYGIH	WIWPYGGSTY	ARGYYWYDY							
HJ8_02D	82.3	93.5	0.35	7	64	68	RASQDVSRGVA	SGRETLYS	QQYFNWPIT	AASGFTIDTRPIH	WIWPYGGSTY	ARGRYYYDY							
HJ8_02E	10.1	58.6	0.26	7	64	59	RASQDVEEAVA	DENVRLYS	QQYFNWPIT	AASGFTIRMSTIH	SIWPSRGYTT	ARGYYWYDY							
HJ8_02F	81.7	93.0	0.28	3	29	39	RASQDVSGGVA	SGSPWLYS	QQYFRWPVT	AASGFTIDNYGIH	SIWPSRGYTT	ARGYYWYDY							
HJ8_03C	78.7	92.7	0.44	8	2	14	RASQDVNDGVA	SDKRGLYS	QQYFNWPIT	AASGFTIDNYGIH	SIWPSRGYTT	ARGYYWYDY							

HJ8_03H	73.3	80.0	0.57	5	75	66	RASQDVSRGVA	SGSPWLYS	QQYFRWPIT	AASGFTIDNYGIH	SIFPRSGFTL	ARGRYWYDY						
HJ8_04D	56.5	76.7	0.52	7	13	8	RASQDVGDTVA	DENVRLYS	QQYFRWPVT	AASGFTIDTRPIH	SIFPRPGYTL	ARGYYWYDY						
HJ8_04H	99.0	78.7	0.52	9	3	7	RASQDVGDTVA	SDKRGLYS	QQYFRWPVT	AASGFTIDTRPIH	SIFPRSGFTL	ARGYYWYDY						
HJ8_05H	28.5	74.6	0.50	5	82	84	RASQDVNDGVA	SGSPWLYS	QQYFRWPVT	AASGFTIDTRPIH	SIWPSRGYTT	ARGYYWYDY						
HJ8_06D	94.0	96.5	0.17	6	22	22	RASQDVNDGVA	SDKRGLYS	QQYFRWPVT	AASGFTIDNYGIH	WIWPYGGSTY	ARGYYWYDY						
HJ8_07H	44.9	68.9	0.51	9	49	51	RASQDVNDGVA	SDKRGLYS	QQYFNWPVT	AASGFTIDTRPIH	SIWPSRGYTT	ARGYYWYDY						
HJ8_08B	11.1	46.4	0.27	5	83	87	RASQDVNDGVA	GRNDILYS	QQYFNWPIT	AASGFTIDNYGIH	SIFPRSGFTL	ARGYYWYDY						
HJ8_11C	38.7	57.0	0.50	7	55	83	RASQDVSRGVA	GRNDILYS	QQYFNWPIT	AASGFTIDNYGIH	SIFPRSGFTL	ARGRYWYDY						
CPT_003	84.2	82.7	-0.04	1	34	26	RASQDVSRGVA	SGSPWLYS	QQYFRWPVT	AASGFTIDNYGIH	WIWPYGGSTY	ARGYYWYDY						
CPT_016	87.3	84.4	0.41	5	7	11	RASQDVSRGVA	SGSPWLYS	QQYFRWPVT	AASGFTIRMSTIH	SIFPRSGFTL	ARGRYYYDY						
CPT_024	15.7	60.4	0.39	4	69	82	RASQDVEEAVA	SGSPWLYS	QQYFNWPIT	AASGFTIDTRPIH	SIWPSRGYTT	ARGYYWYDY						
CPT_026	100.2	93.9	0.11	3	3	7	RASQDVSGGVA	SGSPWLYS	QQYFRWPVT	AASGFTIRMSTIH	SIWPLRGTTS	ARGYYWYDY						
CPT_030	94.6	90.8	0.55	9	17	34	RASQDVSRGVA	SDKRGLYS	QQYFNWPVT	AASGFTIDNYGIH	SIFPRSGFTL	ARGRYWYDY						
CPT_042	84.3	87.1	0.83	11	0	8	RASQDVSRGVA	SDKRGLYS	QQYFRWPVT	AASGFTIDNYGIH	SIWPSRGYTT	ARGRYYYDY						
CPT_071	22.6	48.8	0.15	3	52	71	RASQDVNDGVA	SGSPWLYS	QQYFRWPVT	AASGFTIRMSTIH	SIFPRSGFTL	ARGYYWYDY						
CPT_075	82.8	74.8	0.04	5	1	3	RASQDVSRGVA	GRNDILYS	QQYFNWPIT	AASGFTIRMSTIH	WIWPYGGSTY	ARGRYYYDY						
CPT_095	88.5	76.3	-0.17	0	21	11	RASQDVSRGVA	SGSPWLYS	QQYFNWPIT	AASGFTIDNYGIH	WIWPYGGSTY	ARGYYWYDY						
CPT_111	11.3	50.6	0.40	5	64	92	RASQDVNDGVA	SGSPWLYS	QQYFRWPVT	AASGFTIRMSTIH	SIFPRSGFTL	ARGRYWYDY						
CPT_120	108.2	93.8	0.41	5	3	7	RASQDVSRGVA	SGSPWLYS	QQYFRWPVT	AASGFTIRMSTIH	SIFPRSGFTL	ARGRYYYDY						
CPT_124	69.2	81.6	-0.28	0	85	76	RASQDVSGGVA	SGSPWLYS	QQYFRWPVT	AASGFTIRMSTIH	WIWPYGGSTY	ARGYYWYDY						
CPT_130	28.4	56.9	0.08	5	76	75	RASQDVSRGVA	DENVRLYS	QQYFNWPIT	AASGFTIDTRPIH	WIWPYGGSTY	ARGYYWYDY						
CPT_138	14.2	51.4	0.22	3	56	85	RASQDVNDGVA	SGSPWLYS	QQYFRWPVT	AASGFTIDNYGIH	WIWPYGGSTY	ARGRYWYDY						
CPT_139	84.9	80.5	0.37	5	2	5	RASQDVNDGVA	SGSPWLYS	QQYFNWPIT	AASGFTIRMSTIH	SIWPSRGYTT	ARGRYYYDY						
CPT_159	85.7	74.1	-0.15	1	29	29	RASQDVSRGVA	SGSPWLYS	QQYFNWPIT	AASGFTIRMSTIH	WIWPYAGSTY	ARGRYWYDY						
YK-03	97.3	80.1	0.38	5	19	24	RASQDVSRGVA	SGSPWLYS	QQYFRWPVT	AASGFTIRMSTIH	SIFPRSGFTL	ARGRYWYDY						
YK-06	99.1	99.7	0.51	5	2	15	RASQDVSGGVA	SGSPWLYS	QQYFRWPVT	AASGFTIDTRPIH	SIWPSRGYTT	ARGTYWYDY						
YK-08	88.9	100.0	0.00	2	21	25	RASQDVSRGVA	SGSPWLYS	QQYFNWPIT	AASGFTIRMSTIH	SIFPRSGFTL	ARGYYWYDY						

YK-10	99.6	100.1	-0.19	1	26	24	RASQDVSRGVA	SGSPWLYS	QQYFRWPVT	AASGFTIRMSTIH	WIWPYGGSTY	ARGYYWYDY							
YK-18	96.5	98.6	0.00	2	7	27	RASQDVSRGVA	SGSPWLYS	QQYFNWPIT	AASGFTIRMSTIH	SIFPRSGFTL	ARGYYWYDY							
YK-32	57.8	73.8	0.86	11	34	57	RASQDVNDGVA	SDKRGLYS	QQYFRWPVT	AASGFTIDTRPIH	SIFPRSGFTL	ARGRYWYDY							
YK-36	84.4	86.1	0.75	10	4	11	RASQDVSGGVA	SDKRGLYS	QQYFRWPVT	AASGFTIDNYGIH	SIWPSRGYTT	ARGRYYYDY							
YK-41	91.3	88.5	0.67	10	5	16	RASQDVSRGVA	SDKRGLYS	QQYFRWPVT	AASGFTIDTRPIH	SIWPSRGYTT	ARGYYWYDY							
YK-43	99.2	81.6	0.56	10	7	9	RASQDVSGGVA	SDKRGLYS	QQYFRWPVT	AASGFTIRMSTIH	SIWPSRGYTT	ARGRYWYDY							
YK-72	45.1	65.6	0.38	4	57	82	RASQDVNDGVA	SGSPWLYS	QQYFRWPVT	AASGFTIDNYGIH	SIWPSRGYTT	ARGYYWYDY							
YK-73	90.8	86.9	-0.10	4	37	42	RASQDVSRGVA	GRNDILYS	QQYFRWPVT	AASGFTIRMSTIH	WIWPYGGSTY	ARGYYWYDY							
YK-74	98.3	80.4	0.95	12	1	14	RASQDVSRGVA	SDKRGLYS	QQYFRWPVT	AASGFTIDTRPIH	SIWPSRGYTT	ARGRYYYDY							
YK-82	103.2	80.5	0.47	11	11	17	RASQDVGDTVA	SGRETLYS	QQYFRWPVT	AASGFTIRMSTIH	SIWTSRGYTT	ARGRYWYDY							
YK-89	96.9	72.4	0.75	10	40	57	RASQDVSRGVA	SGRETLYS	QQYFRWPVT	AASGFTIDNYGIH	SIWPSRGYTT	ARGRYWYDY							
YK-92	10.3	43.9	0.00	2	78	97	RASQDVNDGVA	GNYGLLYS	QQYFNWPIT	AASGFTIDNYGIH	WIWPYGGSTY	ARGRYWYDY							
YK-93	103.5	82.9	0.60	10	18	53	RASQDVSRGVA	SGRETLYS	QQYFRWPVT	AASGFTIRMSTIH	SIWPSRGYTT	ARGRYWYDY							
YK-94	92.8	85.3	0.15	2	24	37	RASQDVSGGVA	SGSPWLYS	QQYFNWPIT	AASGFTIDNYGIH	SIWPSRGYTT	ARGYYWYDY							
YK-97	85.8	76.4	0.86	11	56	84	RASQDVSRGVA	SGRETLYS	QQYFRWPVT	AASGFTIDTRPIH	SIWPSRGYTT	ARGRYWYDY							
CY1-A04	112.8	117.5	0.84	12	7	2	RASQDVSDGVA	GREDLLYS	QQYFNFPTT	AASGFTIENRPIH	LISPLRGHTT	ARGRYYYDY							
CY1-A06	111.3	109.0	0.60	-1	13	4	RASQDVPEAVA	STPIRLYS	QQYFRWPTT	AASGFTIDAMPIH	AIYPRAGITL	ARGYYWLDY							
CY1-A08	19.0	55.2	0.49	3	79	78	RASQDVQDGVA	GLKGLLYS	QQYFRWPVT	AASGFTINNQAIH	MIWPRGGVTV	ARARYWLDY							
CY1-A10	13.5	56.6	0.34	4	86	79	RASQDVQDAVA	NGGNSLYS	QQYFRWPVT	AASGFTIDNYGIH	LIWPFKGYTL	ARGKYWYDY							
CY1-B12	22.1	60.3	0.40	9	63	68	RASQDVEEGVA	DEGKRLYS	QQYFNWPVT	AASGFTIRRGAIH	WIWPYGGSTY	ARGSYWLDY							
CY1-C01	15.3	58.5	0.84	6	66	71	RASQDVGGGVA	GWNNTLYS	QQYSRWPAT	AASGFTINKSSIH	AIWPTRGITI	ARGRYWYDY							
CY1-D04	27.2	90.0	0.56	4	41	22	RASQDVSDAVA	GVQPLLYS	QQYNRWPTT	AASGFTIHQSAIH	SIVPSSGITL	ARGRYFYDY							
CY1-D10	17.4	61.4	1.03	7	75	65	RASQDVTDGVA	DQTNLLYS	QQYLRWPST	AASGFTISAKPIH	SIYPRTGFTL	ARGSYWQDY							
CY1-D11	24.2	51.7	0.16	0	74	72	RASQDVGDAVA	SNPTLLYS	QQYNTWPTT	AASGFTITSTPIH	LIWPIRGYTY	ARATYWLDY							
CY1-E06	42.7	53.5	1.04	11	59	57	RASQDVLDGVA	DRRDILYS	QQYYRWPTT	AASGFTIRTKPIH	SIGPSKGLTL	ARGLYWQDY							
CY1-E10	114.7	84.8	1.20	7	6	1	RASQDVSDGVA	SQLELLYS	QQYNRWPST	AASGFTINHNPIH	SIRPRAGITL	ARGAYWLDY							
CY1-E11	56.2	65.3	0.48	4	65	75	RASQDVGGGVA	GAFQSLYS	QQYHSWPIT	AASGFTIPSQSIH	SIYPTRGATL	ARGRFWLDY							

CY1-F08	91.5	99.8	0.28	3	4	3	RASQDVEAGVA	SGSPWLYS	QQYYEWPST	AASGFTIKTKVIH	TIWPWKGFTL	ARGYYWYDY							
CY1-G05	100.3	93.9	0.59	4	48	32	RASQDVMEGVA	SGSPWLYS	QQHNTWPVT	AASGFTIDSWPIH	NIWPFKGYTM	ARGRYWLDY							
CY1-H07	113.2	93.7	0.48	2	3	0	RASQDVNDGVA	RHGPPLYS	QQYYQWPST	AASGFTILSGPIH	SIYPYKGMTL	ARGYYWYDY							
CY2-A10	23.3	53.6	0.62	11	63	61	RASQDVEVGVA	DETKRLYS	QQYFNWPVT	AASGFTIRHTPIH	SINPRRGFTL	ARGQYWLDY							
CY2-A12	12.7	35.4	0.70	8	75	80	RASQDVTEGVA	DRDKVLYS	QQYFRWPVT	AASGFTIPDHPIH	DIFPKAGMTL	ARGLYWLDY							
CY2-B01	7.8	49.4	0.69	6	72	78	RASQDVGDAVA	SRNDLLYS	QQYFRWPVT	AASGFTIGASPIH	TIYPKRGITA	ARGRYWLDY							
CY2-C05	6.7	46.1	0.74	5	91	86	RASQDVMESVA	GVNNLLYS	QQYNNWPTT	AASGFTIRMSPIH	SIWPRRGFTI	ARGRYWLDY							
CY2-C06	68.0	80.9	-0.03	1	74	71	RASQDVMEGVA	SQALRLYS	QQYFNWPIT	AASGFTINLYAIH	SIFPQKGVTT	ARGSYWLDY							
CY2-C09	6.3	39.2	0.49	8	92	87	RASQDVTDGVA	DRDTWLYS	QQYFRWPVT	AASGFTIHRMPIH	AIFPGRGMTL	ARGYYWHDY							
CY2-D03	7.6	41.2	-0.11	2	85	81	RASQDVSDGVA	DRASVLYS	QQYFNWPVT	AASGFTIHTTAIH	AIYPYRGMTM	ARGYYWLDY							
CY2-D10	10.1	29.3	0.69	6	75	73	RASQDVSDGVA	GWNGHLYS	QQYNNWPTT	AASGFTIGQNAIH	SIYPHAGVTM	ARGRYWVDY							
CY2-E05	19.5	58.2	0.51	9	67	71	RASQDVEVGVA	SEQTRLYS	QQYFKWPVT	AASGFTIRSHGIH	LIRPARGGTV	ARGYYWYDY							
CY2-G09	108.0	83.4	0.74	6	22	23	RASQDVKDGVA	TGTLGLYS	QQHNSWPST	AASGFTIPAVGIH	SIFPGNGLTM	ARGRFWLDY							
CY2-H05	18.7	56.5	0.52	7	67	82	RASQDVAEAVA	DERYRLYS	QQYNNWPTT	AASGFTIGSATIH	TIYPTTGFTL	ARGRYWLDY							
CY2-H12	82.8	79.4	0.46	10	47	55	RASQDVDGAVA	SENDRLYS	QQYFRWPVT	AASGFTISVTPIH	LILPRRGNTV	ARGRYWEDY							
HS1A-08	69.8	89.0	0.03	-1	53	42	RASQDVATAVA	GIGETLYS	QQYFNWPIT	AASGFTILDGPIH	WIWPYGGSTY	ARGAYWQDY							
HS1A-09	60.8	80.5	0.91	11	12	15	RASQDVGEGVA	DRRDVLYS	QQHNTWPVT	AASGFTIARSPIH	SIFPAKGHTI	ARGQYWLDY							
HS1A-13	27.9	66.4	0.80	9	70	72	RASQDVLDGVA	GYHDLLYS	QQYFRWPST	AASGFTIRNNSIH	LIWPKKGSTL	ARGRFWLDY							
HS1A-14	7.8	42.7	0.00	0	70	77	RASQDVMEGVA	GWNNLLYS	QQYFRWPST	AASGFTINLQPIH	SIWPSIGITT	ARGFYWLDY							
HS1X-03	76.9	94.5	0.30	1	50	56	RASQDVGKGVA	GYGGYLYS	QQYFRWPAT	AASGFTIATTPIH	SIYPGAGFTL	ARGRYFYDY							
HS1X-04	57.5	90.0	0.71	8	17	10	RASQDVGEDVA	DRQGILYS	QQYFRWPVT	AASGFTITGKGIH	AIYPYKGMTM	ARGRYWVDY							
HS1X-05	52.2	66.4	0.81	6	46	50	RASQDVEDAVA	NHPIGLYS	QQYYRWPTT	AASGFTILQGPIH	SIWPSRGVTT	ARGRYYYDY							
HS1X-06	7.3	37.5	0.46	8	73	89	RASQDVEGGVA	DRDEILYS	QQYFNWPTT	AASGFTISNRPIH	WIWPYGGSTY	ARGRYYYDY							
HS1X-08	54.4	71.7	0.07	6	45	38	RASQDVGVGVA	DRGYFLYS	QQHYRWPTT	AASGFTIFSHPIH	LIWPHRGATM	ARGTYWWDY							
HS1X-10	55.9	75.8	-0.08	3	71	84	RASQDVKDGVA	NNVVNLYS	QQYFNWPVT	AASGFTITNKAIH	SIFPHYGVTL	ARGQYWLDY							
HS1X-11	15.3	58.4	0.07	8	85	76	RASQDVEEGVA	GWQSHLYS	QQYFSWPVT	AASGFTISDSVIH	FIFPKRGSTI	ARGRYWVDY							
HS1X-12	18.4	50.5	0.17	2	93	86	RASQDVGAGVA	DRINKLYS	QQYFNWPIT	AASGFTIDMSAIH	LIWPPLGNTV	ARGRYWLDY							

HS1X-13	74.9	71.3	-0.16	3	77	72	RASQDVSDAVA	DQTFLLYS	QQYNRWPST	AASGFTITKYTIH	LIWPWRGMTV	ARGYYWYDY							
HS1X-15	7.8	29.1	0.39	6	67	77	RASQDVSGGVA	DRDRYLYS	QQYFRWPST	AASGFTIGSIPIH	SIFPLRGFTT	ARGYYWYDY							
HS1X-21	93.0	68.0	0.98	10	26	35	RASQDVDDGVA	GMKGLLYS	QQYNRWPTT	AASGFTITGQTIH	GIYPRKGITI	ARGRYIYDY							
HS1X-23	7.3	57.6	0.23	3	98	100	RASQDVSEGVA	GAFHQLYS	QQYFRWPVT	AASGFTIPGWPIH	NIYPSRGFTL	ARGKYWFDY							
HS1X-25	56.8	90.3	0.22	5	71	61	RASQDVGRGVA	GKNPLLYS	QQYFIWPVT	AASGFTISHNTIH	SIYPPRGFTL	ARGRYWQDY							
HS1X-31	85.0	92.4	-0.08	2	73	69	RASQDVNDGVA	GMNMLLYS	QQYFNWPIT	AASGFTIVSATIH	MIKPVKGKTV	ARGLYWQDY							
HS2A-03	68.5	77.4	0.40	6	54	57	RASQDVGGGVA	DRNTVLYS	QQYYRWPVT	AASGFTIERSAIH	SIYPYKGVTS	ARGYYWYDY							
HS2A-06	68.3	60.4	0.95	10	74	65	RASQDVNEGVA	DERMRLYS	QQYFRWPTT	AASGFTINANTIH	DIFPRSGFTL	ARARYWYDY							
HS2A-07	95.1	69.9	0.84	9	28	21	RASQDVNDGVA	NQMTSLYS	QQYNSWPST	AASGFTISTNTIH	SIHPKQGVTL	ARGRYWYDY							
HS2A-09	16.8	61.3	0.60	4	91	90	RASQDVQDAVA	GAWSQLYS	QQYLRWPTT	AASGFTIRDNPIH	LIFPSSGATV	ARGRYFYDY							
HS2A-12	12.7	58.6	0.10	0	87	90	RASQDVMEGVA	SNPRLLYS	QQYATWPVT	AASGFTISRVPIH	WIWPYGGSTY	ARGTYWMDY							
HS2A-16	16.3	48.4	0.60	8	90	81	RASQDVQEAVA	DQTKMLYS	QQYYRWPVT	AASGFTISANTIH	LIYPNRGHTV	ARGRFWQDY							
HS2X-06	43.8	46.5	0.49	5	66	71	RASQDVGKGVA	AMQDTLYS	QQYFRWPVT	AASGFTIRTTTIH	SIFPVRGYTM	ARARYWLDY							
HS2X-07	8.5	33.6	0.20	6	66	78	RASQDVEDGVA	DRNRHLYS	QQYFNWPVT	AASGFTILGAPIH	SIYPIRGWTT	ARGYYWYDY							
HS2X-09	7.0	32.2	0.22	5	71	49	RASQDVEDGVA	DRIVQLYS	QQYFNWPIT	AASGFTILGNTIH	SIWPRSGITM	ARGLYWQDY							
HS2X-10	27.5	50.4	0.55	3	66	74	RASQDVNNAVA	GVDNLLYS	QQYNNWPTT	AASGFTIATTTIH	SIGPYRGLTL	ARKMYFMDY							
HS2X-13	5.4	46.2	0.68	5	94	95	RASQDVKSAVA	SGNLGLYS	QQYFRWPST	AASGFTIVRMPIH	SIFPHRGFTL	ARGRYWLDY							
HSA1_17	49.3	89.3	0.74	10	57	48	RASQDVRRGVA	DESLRLYS	QQYFRWPVT	AASGFTIRPDPIH	SIHPWRGVTG	ARGYFWLDY							
HSA1_21	6.2	36.5	0.40	8	78	85	RASQDVKEGVA	DQTTRLYS	QQYFNWPVT	AASGFTILTSPIH	SIWPRRGVTL	ARGRYWIDY							
HSA2_17	8.5	41.4	0.55	3	75	82	RASQDVNDAVA	NGSLGLYS	QQYFRWPTT	AASGFTIQRPAIH	SIWPGQGVTT	ARGYYWYDY							
HSA2_18	15.5	53.8	0.16	2	70	74	RASQDVTGGVA	GWGGLLYS	QQYHTWPTT	AASGFTIEAKAIH	SIYPGAGVTM	ARGYYWYDY							
HSA2_19	5.6	37.8	-0.12	0	71	85	RASQDVTNAVA	GAFNGLYS	QQYYRWPVT	AASGFTISSNVIH	WIWPYGGSTY	ARGRFWLDY							
HSA2_20	17.9	55.4	0.46	3	70	71	RASQDVNDGVA	PRATYLYS	QQYYRWPVT	AASGFTIGNRPIH	SIWPLLGITT	ARGRYWYDY							
HSA2_23	8.1	40.3	0.67	8	72	86	RASQDVTGGVA	GAFQQLYS	QQYHNWPTT	AASGFTISQRAIH	SIYPRVGVTS	ARGRYWHDY							
HSX1_36	55.8	78.5	0.77	7	55	57	RASQDVADGVA	GLHQLLYS	QQYSRWPAT	AASGFTIKDRSIH	SIYPASGFTL	ARGKYWYDY							
HSX1_37	12.9	46.9	0.61	7	80	90	RASQDVAEGVA	DRDRYLYS	QQYYRWPST	AASGFTIGTNPIH	VIWPKRGLTI	ARGYYWLDY							

HSX1_42	79.6	111.2	0.16	-1	52	62	RASQDVLNAVA	GANGMLYS	QQYFNWPVT	AASGFTISNEPIH	SIWPQKGLTV	ARGYYWLDY							
HSX1_46	23.3	77.3	0.48	7	45	50	RASQDVGDGVA	GVQRLLYS	QQYNTWPTT	AASGFTIYLNTIH	SIWPKSGFTL	ARGRYWEDY							
HSX1_47	106.8	123.7	0.06	5	3	3	RASQDVSGSVA	TRVTHLYS	QQYSSWPTT	AASGFTIHHLPIH	SIYPIKGFTL	ARGYYWYDY							
HSX1_48	106.9	100.0	0.55	2	0	12	RASQDVSNAVA	GYNGLLYS	QQYHSWPST	AASGFTISHPAIH	TIMPSSGITL	ARRMYVMDY							
HSX2_21	100.2	86.5	0.27	7	33	36	RASQDVVEGVA	DRNIILYS	QQYNTWPVT	AASGFTIDDKTIH	LIMPKKGFTV	ARGYYWLDY							
HSX2_22	9.4	45.6	1.08	11	72	84	RASQDVEDGVA	GFHGRLYS	QQYNRWPTT	AASGFTIGRNTIH	SIFPMRGFTL	ARGRFWLDY							
HSX2_28	5.3	45.0	0.88	5	75	88	RASQDVADGVA	GAQRQLYS	QQYNNWPTT	AASGFTINTSAIH	SIFPLKGLTM	ARGRYWYDY							
HSX2_31	83.3	77.4	0.84	9	54	58	RASQDVADGVA	GWDGLLYS	QQYHNWPTT	AASGFTISRHSIH	LIRPKSGITV	ARGGYYLDY							
HJ1_03F	100.3	81.3	0.48	5	40	48	RASQDVSRGVA	SGSPWLYS	QQYFRWPVT	AASGFTIDTRPIH	SIWPSRGYTT	ARGYYWYDY							
HJ1_05A	103.3	71.4	0.81	10	38	27	RASQDVSGGVA	SGRETLYS	QQYFRWPVT	AASGFTIDTRPIH	SIWPSRGYTT	ARGRYYYDY							
HJ6_10D	8.9	42.7	0.64	9	88	90	RASQDVNDGVA	DENVRLYS	QQYFNWPIT	AASGFTIDNYGIH	SIWPSRGYTT	ARGRYWYDY							
HJ7_02F	105.8	98.3	0.66	10	6	27	RASQDVEEAVA	SDKRGLYS	QQYFRWPVT	AASGFTIRMSTIH	SIWPTRGYTT	ARGYYCYDY							
HJ7_09E	83.7	79.0	0.40	8	68	94	RASQDVNDGVA	SGRETLYS	QQYFNWPIT	AASGFTIRMSTIH	SIFPRSGFTL	ARGRYWYDY							
HJ7_12D	58.4	70.5	0.64	10	69	79	RASQDVEEAVA	SGRETLYS	QQYFRWPVT	AASGFTIRMSTIH	SIWPSRGYTT	ARGRYWYDY							
HJ8_04F	39.6	56.5	0.19	6	80	72	RASQDVNDGVA	GRNDILYS	QQYFNWPIT	AASGFTIRMSTIH	SIWPSRGYTT	ARGYYWYDY							
HJ8_10H	103.6	66.9	0.81	10	62	78	RASQDVSRGVA	SGRETLYS	QQYFRWPVT	AASGFTIDTRPIH	SIFPRSGFTL	ARGRYYYDY							
CPT_027	103.6	87.6	0.67	6	2	24	RASQDVSGGVA	SGSPWLYS	QQYFRWPVT	AASGFTIDTRPIH	SIWPSRGYTT	ARGRYYYDY							
CPT_039	104.6	111.1	0.59	9	3	27	RASQDVSGGVA	SDKRGLYS	QQYFRWPVT	AASGFTIDTRPIH	SIWPSRGYTT	ARGYYWYDY							
CPT_048	52.9	82.1	0.69	9	82	85	RASQDVNDGVA	SGRETLYS	QQYFRWPVT	AASGFTIDNYGIH	SIFPRSGFTL	ARGRYWYDY							
CPT_090	90.8	115.0	0.59	8	25	43	RASQDVNDGVA	DENVRLYS	QQYFNWPIT	AASGFTIDNYGIH	SIFPRSGFTL	ARGRYYYDY							
CPT_094	104.1	93.7	0.53	9	2	20	RASQDVNDGVA	NDNRGLYS	QQYFNWPIT	AASGFTIRMSTIH	SIWPSRGYTT	ARGRYYYDY							
CPT_103	104.7	99.0	0.26	8	2	17	RASQDVGDTVA	SDKRGLYS	QQYFRWPVT	AASGFTIRMSTIH	SIFPRSGFTL	ARGYYWYDY							
CPT_106	101.7	79.0	0.24	7	3	19	RASQDVNDGVA	DENVRLYS	QQYFRWPVT	AASGFTIRMSTIH	WIWPYGGSTY	ARGRYYYDY							
CPT_108	91.1	69.9	0.72	6	51	72	RASQDVEEAVA	SGSPWLYS	QQYFRWPVT	AASGFTIDTRPIH	SIFPRSGFTL	ARGRYYYDY							
CPT_112	62.2	77.2	0.52	7	81	81	RASQDVNDGVA	GRNDILYS	QQYFRWPVT	AASGFTIDTRPIH	SIFPRSGFTL	ARGYYWYDY							
CPT_119	83.4	89.7	0.54	6	73	76	RASQDVGDTVA	SGSPWLYS	QQYFRWPVT	AASGFTIDNYGIH	SIWPSRGYTT	ARGRYWYDY							

CPT_128	67.2	87.3	0.66	9	43	40	RASQDVSRGVA	DENVRLYS	QQYFNWPIT	AASGFTIDTRPIH	SIFPRSGFTL	ARGRYWYDY						
CPT_134	101.0	97.0	0.42	8	14	31	RASQDVNDGVA	SDKRGLYS	QQYFNWPIT	AASGFTIDTRPIH	WIWPYGGSTY	ARGRYYYDY						
CPT_140	37.9	60.4	0.53	9	65	67	RASQDVSRGVA	DENVRLYS	QQYFRWPVT	AASGFTIDNYRIH	SIWPSRGYTT	ARGYYWYDY						
CPT_142	95.6	84.4	0.84	10	19	26	RASQDVNDGVA	DENVRLYS	QQYFRWPVT	AASGFTIDTRPIH	SIFPRSGFTL	ARGRYYYDY						
CPT_149	102.8	86.7	0.35	7	9	31	RASQDVSRGVA	GRNDILYS	QQYFNWPIT	AASGFTIRMSTIH	SIFPRSGFTL	ARGRYWYDY						
CPT_156	99.7	72.9	0.60	10	32	45	RASQDVSRGVA	DENVRLYS	QQYFRWPVT	AASGFTIRMSTIH	SIWPSRGYTT	ARGRYWYDY						
CPT_160	101.0	105.3	0.14	6	2	17	RASQDVSRGVA	GRNDILYS	QQYFNWPIT	AASGFTIQMSTIH	SIFPRSGFTL	ARGRYYYDY						
YK-02	101.6	102.2	0.56	8	2	16	RASQDVSRGVA	SDKRGLYS	QQYFRWPVT	AASGFTIVPTPIH	SIWPSRGYTT	ARGRYWYDY						
YK-07	84.2	82.8	0.40	7	55	69	RASQDVNDGVA	GRNDILYS	QQYFNWPIT	AASGFTIRMSTIH	SIWPSRGFTL	ARGRYYYDY						
YK-09	100.1	85.4	0.34	8	22	47	RASQDVNDGVA	SDKRGLYS	QQYFRWPVT	AASGFTIRMSTIH	SIFPRSGFTL	ARGYYWYDY						
YK-11	100.7	78.9	0.61	9	39	55	RASQDVNDGVA	SDKRGLYS	QQYFRWPVT	AASGFTIDTRPIH	SIFPRSGFTL	ARGYYWYDY						
YK-12	104.9	88.8	0.30	7	40	59	RASQDVSGGVA	SGRETLYS	QQYFNWPIT	AASGFTIRMSTIH	SIFPRSGFTL	ARGRYWYDY						
YK-14	103.2	75.4	0.45	5	20	37	RASQDVEEAVA	SGSPWLYS	QQYFRWPVT	AASGFTIRMSTIH	SIFPRSGFTL	ARGRYYYDY						
YK-17	97.1	66.5	0.36	7	57	65	RASQDVNDGVA	SDKRGLYS	QQYFNWPIT	AASGFTIDNYGIH	SIFPRSGFTL	ARGYYWYDY						
YK-19	66.7	94.0	0.66	6	77	81	RASQDVEEAVA	SGSPWLYS	QQYFNWPIT	AASGFTIDTRPIH	SIWPSRGYTT	ARGRYYYDY						
YK-23	103.3	95.4	0.33	7	29	51	RASQDVSGGVA	SGRETLYS	QQYFNWPIT	AASGFTIRMSTIH	SIFPRSGFTL	ARGRYYYDY						
YK-24	11.0	41.2	0.45	7	90	91	RASQDVNDGVA	DENVRLYS	QQYFRWPVT	AASGFTIDNYGIH	SIFPRSGFTL	ARGYYWYDY						
YK-28	77.9	82.4	0.29	8	76	68	RASQDVNDGVA	SDKRGLYS	QQYFNWPIT	AASGFTIRMSTIH	SIWPSRGYTT	ARGYYWYDY						
YK-29	103.4	94.0	0.51	8	38	50	RASQDVSRGVA	GRNDILYS	QQYFRWPVT	AASGFTIRMSTIH	SIFPRSGFTL	ARGRYYYDY						
YK-33	22.4	59.5	0.55	5	98	92	RASQDVNDGVA	SGSPWLYS	QQYFRWPVT	AASGFTIDNYGIH	SIFPRSGFTL	ARGRYWYDY						
YK-34	20.9	41.6	0.43	7	92	91	RASQDVSRGVA	DENVRLYS	QQYFRWPVT	AASGFTIDNYGIH	SIFPRSGFTL	ARGYYWYDY						
YK-35	15.5	60.0	0.40	5	97	99	RASQDVNDGVA	GRNDILYS	QQYFRWPVT	AASGFTIVPTPIH	SIFPRSGFTL	ARGRYWYDY						
YK-37	100.6	113.7	0.77	7	39	48	RASQDVNDGVA	SGSPWLYS	QQYFRWPVT	AASGFTIDTRPIH	SIFPRSGSTY	ARGRYYYDY						
YK-39	101.6	95.7	0.49	6	11	25	RASQDVSRGVA	SGSPWLYS	QQYFRWPVT	AASGFTIRMSTIH	SIWPSRGYTT	ARGRYYYDY						
YK-42	23.3	54.0	0.36	7	92	93	RASQDVNDGVA	GRNDILYS	QQYFNWPIT	AASGFTIRMSTIH	SIFPRSGFTL	ARGRYWYDY						
YK-44	89.3	83.3	0.22	7	24	28	RASQDVSRGVA	DENVRLYS	QQYFNWPIT	AASGFTIRMSTIH	SIWPSRGYTT	ARGYYWYDY						
YK-46	103.5	85.1	0.64	6	7	21	RASQDVSRGVA	SGSPWLYS	QQYFRWPVT	AASGFTIDNYGIH	SIWPSRGYTT	ARGRYYYDY						

YK-47	79.8	75.1	-0.05	5	69	69	RASQDVSRGVA	DENVRLYS	QQYFRWPVT	AASGFTIRMSTIH	WIWPYGGSTY	ARGYYWYDY							
YK-52	102.7	77.8	0.23	6	20	38	RASQDVSRGVA	GRNDILYS	QQYFRWPVT	AASGFTIRMSTIH	SIFPRSGFTL	ARGYYWYDY							
YK-54	100.9	100.0	0.58	6	53	64	RASQDVGDTVA	SGSPWLYS	QQYFRWPVT	AASGFTIDTRPIH	SIFPRSGFTL	ARGRYWYDY							
YK-55	44.1	66.7	0.58	8	90	96	RASQDVSRGVA	GRNDILYS	QQYFNWPIT	AASGFTIDNYGIH	SIWPSRGYTT	ARGRYWYDY							
YK-57	102.2	100.0	0.76	7	33	47	RASQDVSRGVA	SGSPWLYS	QQYFRWPVT	AASGFTIDTRPIH	SIWPSRGYTT	ARGRYYYDY							
YK-58	20.8	57.1	0.29	7	82	91	RASQDVNDGVA	SGRETLYS	QQYFRWPVT	AASGFTIRMSTIH	SIFPRSGFTL	ARGYYWYDY							
YK-59	102.5	87.4	0.47	7	31	46	RASQDVSRGVA	GRNDILYS	QQYFRWPVT	AASGFTIDNYGIH	SIWPSRGYTT	ARGYYWYDY							
YK-60	103.0	86.8	0.42	7	9	27	RASQDVSGGVA	SGRETLYS	QQYFRWPVT	AASGFTIDNYGIH	SIWPSRGYTT	ARGYYWYDY							
YK-61	103.0	74.4	0.61	8	40	30	RASQDVSRGVA	GRNDILYS	QQYFNWPIT	AASGFTIDNYGIH	SIWPSRGYTT	ARGRYYYDY							
YK-62	54.6	62.9	0.37	8	59	60	RASQDVNDGVA	DENVRLYS	QQYFRWPVT	AASGFTIRMSTIH	SIWPSRGYTT	ARGYYWYDY							
YK-65	76.2	84.0	0.82	10	85	91	RASQDVSRGVA	GRNDILYS	QQYFRWPVT	AASGFTIDTRPIH	SIWPSRGYTT	ARGRYWYDY							
YK-68	13.0	39.4	0.27	6	86	87	RASQDVSRGVA	GENVRLYS	QQYFRWPVT	AASGFTIRMSTIH	SIFPRSGFTL	ARGYYWYDY							
YK-75	101.6	91.8	0.56	9	10	32	RASQDVSRGVA	SDKRGLYS	QQYFRWPVT	AASGFTIDNYGIH	SIWPSRGYTT	ARGYYWYDY							
YK-79	28.4	53.5	0.67	9	75	73	RASQDVNDGVA	DENVRLYS	QQYFNWPIT	AASGFTIDNYGIH	SIWPSRGYTT	ARGRYYYDY							
YK-80	20.8	39.2	0.30	5	91	89	RASQDVEEAVA	GRNDILYS	QQYFNWPIT	AASGFTIDNYGIH	SIFPRSGFTL	ARGYYWYDY							
YK-81	20.5	43.1	0.60	9	67	69	RASQDVEEAVA	DENVRLYS	QQYFRWPVT	AASGFTIRMSTIH	SIFPRSGFTL	ARGRYYYDY							
YK-91	82.4	72.3	0.41	5	82	48	RASQDVGDTVA	SGSPWLYS	QQYFNWPIT	AASGFTIDNYGIH	SIWPSRGYTT	ARGRYWYDY							
YK-95	31.7	55.7	0.66	9	86	94	RASQDVNDGVA	SGRETLYS	QQYFNWPIT	AASGFTIDNYGIH	SIWPSRGYTT	ARGRYYYDY							
YK-96	98.4	111.6	0.54	5	43	45	RASQDVSGGVA	SGSPWLYS	QQYFNWPIT	AASGFTIDTRPIH	SIWPSRGYTT	ARGRYYYDY							
YCM2-11A	101.4	102.1	0.40	5	6	20	RASQDVSGGVA	SGSPWLYS	QQYFRWPVT	AASGFTIRMSTIH	SIWPSRGYTT	ARGRYYYDY							
YCM3-03D	92.2	87.5	0.66	8	68	86	RASQDVSRGVA	GRNDILYS	QQYFRWPVT	AASGFTIDNYGIH	SIFPRSGFTL	ARGRYYYDY							
YCM3-05C	34.8	61.9	0.36	8	67	65	RASQDVSRGVA	DENVRLYS	QQYFRWPVT	AASGFTIRMSTIH	SIWPSRGYTT	ARGYYWYDY							
YCM3-12H	101.1	88.3	0.31	8	7	19	RASQDVGDTVA	SGRETLYS	QQYFNWPIT	AASGFTIRMSTIH	SIFPRSGFTL	ARGRYWYDY							
YCM4-01B	101.0	86.3	0.05	5	30	25	RASQDVEEAVA	GRNDILYS	QQYFNWPVT	AASGFTIRMSTIH	WIWPYGGSTY	ARGRYYYDY							
YCM4-01F	100.1	77.8	0.48	5	63	25	RASQDVSRGVA	SGSPWLYS	QQYFRWPVT	AASGFTIDTRPIH	SIWPSRGYTT	ARGYYWYDY							
YCM4-04B	74.5	95.6	0.67	8	43	44	RASQDVSGGVA	DENVRLYS	QQYFRWPVT	AASGFTIDTRPIH	SIWPYGGSTY	ARGRYYYDY							

YCM4-04F	40.3	80.3	-0.02	5	60	57	RASQDVSGGVA	DENVRLYS	QQYFNWPIT	AASGFTIRMSTIH	WIWPYGGSTY	ARGRYWYDY							
YCM4-07C	25.1	48.1	0.08	5	94	41	RASQDVSRGVA	DENVRLYS	QQYFNWPIT	AASGFTIDTRPIH	WIWPYGGSTY	ARGYYWYDY							
YCM4-10E	99.6	90.9	0.54	8	33	57	RASQDVSRGVA	SGRETLYS	QQYFRWPVT	AASGFTIDTRPIH	SIFPRSGFTL	ARGYYWYDY							
YCM4-11B	99.5	91.3	-0.04	5	66	62	RASQDVNDGVA	SGRETLYS	QQYFRWPVT	AASGFTIRMSTIH	WIWPYGGSTY	ARGYYWYDY							
YCM4-11E	41.2	78.3	0.34	5	38	38	RASQDVNDGVA	DENVRLYS	QQYFNWPIT	AASGFTIVPTPIH	SIFPRSGFTL	ARGRYYYDY							
HS1A10B12	9.9	51.6	0.71	5	97	89	RASQDVADAVA	GGFGTLYS	QQYFRWPTT	AASGFTINKLPIH	SIYPYKGLTT	ARGRYWQDY							
HS1A11C12	88.6	89.1	0.28	5	39	27	RASQDVSDGVA	DQMNVLYS	QQYFKWPVT	AASGFTIWNRTIH	LIWPRGGTTM	ARGSYFYDY							
HS1X27_C05	45.4	79.6	0.41	5	86	77	RASQDVQDGVA	RLDYGLYS	QQYFRWPST	AASGFTIADKPIH	VIWPIRGYTI	ARGWYWQDY							
HS2A10_B10	21.8	62.4	0.36	5	97	45	RASQDVGEGVA	GYNSLLYS	QQYFNWPVT	AASGFTITHRPIH	SITPYKGMTF	ARGRFWLDY							
HSX2_29	48.2	67.3	-0.19	2	68	59	RASQDVVDAVA	SREDMLYS	QQYTTWPVT	AASGFTIINYPIH	GIFPYKGITT	ARGYYWYDY							
CY1-G06	7.3	51.3	0.63	3	100	90	RASQDVSDAVA	NGTLGLYS	QQYNNWPTT	AASGFTIRSRPIH	SIFPGMGFTL	ARGAYWYDY							
CY1-G07	58.7	80.6	0.28	3	72	56	RASQDVERGVA	SGSPWLYS	QQHNTWPVT	AASGFTISYKPIH	LIWPPLGNTT	ARGYYWYDY							
CY1-H01	99.5	87.7	0.79	9	33	17	RASQDVRGGVA	SGSPWLYS	QQYLNWPTT	AASGFTIDNYGIH	SIWPRRGFTT	ARGRYWHDY							
CY2-A05	6.4	46.7	-0.06	2	83	79	RASQDVLEGVA	GWNNFLYS	QQYYAWPTT	AASGFTIGRFAIH	SIFPHRGFTT	ARGYYWLDY							
CY2-E01	11.3	47.9	0.20	3	82	79	RASQDVEDGVA	GSFGVLYS	QQYHNWPTT	AASGFTILPHPIH	FIWPGKGATW	ARGAYYYDY							
WY1X-01E	101.9	75.8	0.02	3	10	12	RASQDVGGGVA	DYTLGLYS	QQYITWPVT	AASGFTIPRHGIH	LIWPRSGITV	ARGNYYYDY							
WY1A-09D	20.9	52.5	0.38	3	90	80	RASQDVGAGVA	GWDRYLYS	QQYFNWPIT	AASGFTIGRRTIH	SIFPGAGVTL	ARGQYWLDY							
WY1A-10D	94.0	69.5	0.36	3	51	36	RASQDVGGGVA	GVGNRLYS	QQYNTWPVT	AASGFTIESYPIH	LIWPFSGNTI	ARGRYYYDY							
WY2X-06E	18.1	49.2	0.59	3	80	74	RASQDVTDAVA	GYQNVLYS	QQYNNWPTT	AASGFTIPREPIH	LIWPNSGTTI	ARARYWLDY							
WY2X-06G	20.8	55.0	0.37	8	66	67	RASQDVSGGVA	DREDILYS	QQYNSWPST	AASGFTIHAYTIH	SIWPSRGITT	ARGAYWLDY							
WY2X-06H	17.5	48.8	0.43	3	92	92	RASQDVYPAVA	PTGNTLYS	QQYFNWPST	AASGFTIHNNTIH	DIFPTSGFTI	ARGRFWLDY							
WY2A-07A	28.5	56.8	0.22	2	75	74	RASQDVLEGVA	DRNVILYS	QQYSTWPIT	AASGFTINPPRIH	LIWPTSGITV	ARGSYWLDY							
SCC1R-06	71.1	65.3	-0.18	1	83	77	RASQDVWDGVA	GWNNMLYS	QQYFRWPVT	AASGFTISTTTIH	LINPKLGLTV	ARGYYWYDY							
SCC1R-13	58.0	72.3	-0.05	2	48	36	RASQDVSNGVA	GIPGMLYS	QQYFNWPIT	AASGFTITHTSIH	YITPRAGTTV	ARGLYWQDY							
SCC1R-24	22.3	48.5	0.12	0	76	42	RASQDVSSGVA	SGSPWLYS	QQYNIWPVT	AASGFTISRYGIH	SIYPPRGFTL	ARGYYWYDY							

SCC1R-29	98.8	69.4	0.55	2	26	20	RASQDVGKGVA	GVPNLLYS	QQYNNWPIT	AASGFTITQNPIH	LIRPDMGYTV	ARGLYWQDY							
SCC2R-02	15.6	49.4	0.29	4	89	85	RASQDVMEGVA	DRNLFLYS	QQYFNWPIT	AASGFTIDNYGIH	SIWPRLGFTM	ARGRFWLDY							
SCC2R-03	64.1	62.6	0.27	4	70	69	RASQDVMEGVA	TMTSQLYS	QQYFRWPVT	AASGFTINQEPIH	SIWPYRGFTT	ARGTYWFDY							
SCC2R-04	100.8	89.1	-0.10	0	21	23	RASQDVVGGVA	SSTTLLYS	QQYFNWPIT	AASGFTIQDIAIH	MIRPYAGITI	ARGRYWLDY							
SCC2R-11	40.7	63.5	0.13	2	62	51	RASQDVGQGVA	GYSNSLYS	QQYFTWPAT	AASGFTIHQMPIH	SIFPYRGFTL	ARGAYWHDY							
SCC2G-34	18.0	50.9	0.27	0	78	85	RASQDVLDGVA	GIPRLLYS	QQYSTWPTT	AASGFTIYTSPIH	GIYPNNGITL	ARGYYWYDY							
SCC2G-37	7.8	38.6	0.29	4	75	74	RASQDVSGGVA	DRGWVLYS	QQYFNWPIT	AASGFTISTRPIH	SIYPSRGVTM	ARGLYWQDY							
SCC2G-42	13.3	35.1	-0.13	3	88	82	RASQDVAGAVA	DQTVWLYS	QQYFNWPVT	AASGFTISHHPIH	SIWPLRGYTT	ARGRFWLDY							
SCC2G-43	11.8	38.9	0.45	4	83	82	RASQDVVDAVA	GYQQLLYS	QQYYRWPTT	AASGFTINRGTIH	SIWPGLGHTI	ARARYWLDY							
SCC2G-44	9.2	41.1	-0.41	2	87	85	RASQDVGGGVA	GYHPYLYS	QQYFIWPVT	AASGFTIRMHSIH	SIYPQTGFTL	ARGRYWYDY							
SCC2G-45	13.4	43.7	0.55	3	86	85	RASQDVASAVA	GMDGNLYS	QQYFNWPIT	AASGFTISRSPIH	SISPRAGITF	ARGRYWLDY							
SCC2G-48	34.9	57.1	0.22	3	75	75	RASQDVNGAVA	GGWGMLYS	QQYFNWPST	AASGFTITSSGIH	SIRPESGITL	ARGYYWYDY							
PHT1-A03	100.2	66.2	0.43	10	43	43	RASQDVDGTVA	GRDDHLYS	QQYFNWPIT	AASGFTINSATIH	GIFPTRGVTT	ARGRFWMDY							
PHT1-A08	101.2	77.3	0.85	11	3	10	RASQDVRRGVA	AGDFTLYS	QQYNTWPVT	AASGFTITRDTIH	SIGPSRGWTS	ARGRFWLDY							
PHT1-B02	24.0	56.6	-0.24	0	75	67	RASQDVADSVA	GYANLLYS	QQYFNWPVT	AASGFTITYHPIH	SIWPLRGFTT	ARRMYIMDY							
PHT1-B12	17.0	43.0	-0.03	0	91	86	RASQDVSGAVA	GFQGLLYS	QQYNTWPVT	AASGFTINRIPIH	WIWPYGGSTY	ARGRYWLDY							
PHT1-C09	101.5	86.8	0.51	4	4	20	RASQDVEKGVA	GIPEVLYS	QQYNKWPAT	AASGFTIGHIPIH	LIYPYKGFTV	ARGRFWLDY							
PHT1-D03	101.5	89.9	0.63	3	12	14	RASQDVSGGVA	GAWGSLYS	QQYFRWPST	AASGFTIPKSPIH	SIWPKTGITF	ARGGYWLDY							
PHT1-E02	99.4	75.7	0.26	8	12	23	RASQDVTSGVA	DRTVMLYS	QQYNTWPIT	AASGFTIKHTTIH	VIYPRKGMTI	ARGRFWLDY							
PHT1-F02	99.0	61.6	0.53	4	8	19	RASQDVEDGVA	TRNDGLYS	QQYYRWPVT	AASGFTIPMQPIH	LILPKYGNTL	ARGYYWLDY							
PHT1-F03	27.3	49.6	0.94	11	82	80	RASQDVRDGVA	GGFNQLYS	QQYNNWPTT	AASGFTIRHSTIH	SIFPHRGFTM	ARGRYYYDY							
PHT1-F09	100.3	71.9	0.33	4	24	27	RASQDVALAVA	NQQYHLYS	QQYSNWPTT	AASGFTIQGTTIH	GIMPSKGFTL	ARGRYWYDY							
PHT1-H01	99.1	84.9	0.74	9	8	13	RASQDVLDGVA	DQSYWLYS	QQYSRWPTT	AASGFTIKNTTIH	SITPTRGITL	ARGKYWYDY							
PHT1-H06	22.4	38.7	0.75	8	86	81	RASQDVRDAVA	GVKHLLYS	QQYYRWPVT	AASGFTIPTRGIH	SIYPQRGITT	ARGRYWLDY							

PHT1-H11	88.6	71.5	0.43	4	55	68	RASQDVADGVA	SSRQLLYS	QQYNMWPTT	AASGFTITSWPIH	SIWPFKGMTN	ARGRYFYDY							
PHT2-A01	20.4	50.4	0.12	4	83	80	RASQDVGGTVA	GWNNVLYS	QQYFKWPVT	AASGFTIHKSTIH	LILPKRGATV	ARGAYWYDY							
PHT2-C10	100.2	75.3	1.03	12	23	24	RASQDVSKGVA	DRNTLLYS	QQYSRWPTT	AASGFTIHKEPIH	LIWPERGLTI	ARGRYWLDY							
PHT2-D06	6.5	38.5	0.18	3	78	78	RASQDVVDGVA	SQQTLLYS	QQYYRWPST	AASGFTIVGATIH	SIWPGLGMTT	ARGRYWVDY							
PHT2-D10	11.8	36.3	-0.03	0	88	86	RASQDVVEGVA	SMTHLLYS	QQYFNWPIT	AASGFTIPHNPIH	LIWPGRGITV	ARGAYWLDY							
PHT2-F12	30.1	56.2	0.04	1	73	70	RASQDVVDAVA	GWNNLLYS	QQYNTWPVT	AASGFTISDYTIH	SIYPSGGFTL	ARRMYYLDY							
PHT2-G09	5.6	34.5	0.20	3	82	86	RASQDVTNAVA	SSMSTLYS	QQYFKWPVT	AASGFTIAGHTIH	SIYPMRGMTM	ARGRYWVDY							
PHT2-H05	9.0	42.6	0.90	8	80	83	RASQDVFDGVA	DRNYMLYS	QQYSRWPTT	AASGFTINRASIH	SIWPMRGVTF	ARGRYWQDY							
PHT1-H05	86.8	71.4	0.48	4	70	62	RASQDVGEGVA	GRASLLYS	QQYFNWPIT	AASGFTIDTKPIH	SIWPYNGVTG	ARGSYWLDY							
PHT1-H12	80.3	59.7	0.16	1	71	68	RASQDVQRAVA	SSQRLLYS	QQYSSWPVT	AASGFTISKLPIH	LIWPTQGYTI	ARGYYWLDY							
PHT2-A07	21.3	43.4	0.34	8	81	83	RASQDVTEGVA	DRDRILYS	QQYFNWPIT	AASGFTIDNYGIH	LIRPSSGITT	ARGYYWYDY							
PHT2-C04	8.7	38.7	0.14	-1	88	88	RASQDVAEGVA	GWNAYLYS	QQYFNWPIT	AASGFTIRNYPIH	AIYPGKGFTL	ARGSYWLDY							
PHT2-D04	35.3	54.8	0.43	4	74	73	RASQDVSGGVA	DRTLYLYS	QQYTRWPST	AASGFTITGVPIH	GIWPGAGTTL	ARGTYWLDY							
YC1-01A	99.4	75.4	0.65	9	62	68	RASQDVGGTVA	GWHELLYS	QQYFRWPVT	AASGFTIGKRPIH	TIKPKSGITM	ARGRYWMDY							
YC1-04C	99.2	71.4	0.27	1	26	47	RASQDVGRGVA	ASKGVLYS	QQYNTWPVT	AASGFTIQQAAIH	SIFPPKGVTL	ARGYYWLDY							
YC1-04G	7.2	47.5	0.46	0	83	84	RASQDVTDGVA	GAFAKLYS	QQYFNWPTT	AASGFTIPESPIH	LIFPPRGITL	ARGAYWLDY							
YC1-08A	14.5	47.0	-0.07	0	84	80	RASQDVEAGVA	GIANTLYS	QQYNTWPVT	AASGFTINLSPIH	WIWPYGGSTY	ARGRYWLDY							
YC1-08C	98.7	83.1	0.66	9	5	13	RASQDVGVGVA	DRNWILYS	QQYLKWPTT	AASGFTIDRETIH	SIFPKSGLTL	ARGRYWYDY							
YC1-09B	14.8	48.1	-0.37	-2	74	67	RASQDVGGAVA	GWNYYLYS	QQYFNWPIT	AASGFTIPRDPIH	SIYPVKGFTF	ARGLYWLDY							
YC1-09C	97.5	66.7	0.45	8	16	20	RASQDVEGGVA	DRDTFLYS	QQYFRWPVT	AASGFTIDNVAIH	TINPRMGYTF	ARGRYYYDY							
YC1-09D	15.9	61.7	0.51	8	67	77	RASQDVLEGVA	DQSTRLYS	QQYFNWPIT	AASGFTITNNAIH	SIFPTQGRTS	ARGRFWLDY							
YC1-09G	90.3	71.5	0.79	11	8	23	RASQDVGGGVA	DRQEYLYS	QQHYRWPTT	AASGFTIKQSPIH	TIFPKRGWTM	ARGTYWFDY							
YC1-09H	53.6	63.2	0.43	9	70	63	RASQDVGKDVA	DRDTFLYS	QQYSTWPIT	AASGFTIYNSTIH	SIFPRRGYTM	ARGLYCQDY							
YC1-12H	93.8	72.9	0.62	9	4	14	RASQDVREGVA	DMESRLYS	QQYFNWPIT	AASGFTINHEPIH	SITPRSGLTL	ARGYYWLDY							
YC2-01E	23.3	38.8	0.67	8	85	88	RASQDVTDGVA	GTKNLLYS	QQYFRWPVT	AASGFTIPHHPIH	SILPREGMTF	ARGRFWLDY							
YC2-02B	17.2	46.5	0.45	4	85	84	RASQDVPEAVA	GWNNYLYS	QQYLKWPIT	AASGFTIDRQTIH	LIWPWRGSTQ	ARGAYWQDY							

YC2-03G	14.6	46.4	0.46	9	82	81	RASQDVGRGVA	DRNDKLYS	QQYFNWPST	AASGFTIVERTIH	WIWPYGGSTY	ARGSYWYDY							
YC2-07E	16.0	44.5	0.29	1	83	84	RASQDVSNSVA	GWNYLLYS	QQYNNWPTT	AASGFTISNAAIH	TIWPGAGTTL	ARGRYWLDY							
YC2-10F	8.6	56.1	0.32	4	82	84	RASQDVNGGVA	NMTLGLYS	QQHNTWPIT	AASGFTIGQSAIH	LIWPPKGTTL	ARGRFWLDY							
YC2-11B	21.1	57.0	0.43	4	91	92	RASQDVTDAVA	GWDNRLYS	QQYFRWPVT	AASGFTIAGTPIH	SIWPRVGSTY	ARGTYWLDY							
YC2-11E	16.5	54.4	0.31	8	70	78	RASQDVDDGVA	DRNYVLYS	QQYFNWPVT	AASGFTISDRVIH	LIWPSKGHTI	ARGQYWLDY							
YC2-12E	7.6	41.4	0.40	9	82	87	RASQDVTGGVA	DRTFYLYS	QQYFRWPVT	AASGFTIDRTAIH	SIFPRRGITT	ARGKYWYDY							

Supplementary Table S2. Effects of anti-MSLN IgG1-vc-MMAEs on serum biochemical parameters in N87 NOD/SCID mice. Experimental details are shown in Supplementary Methods.

N87	*ALT(U/L)*	*ALP(U/L)*	*BUN(mg/dL)*	*CRE(mg/dL)*	*ALT/ALP*
PBS	38.17± 9.21	143.67 ± 37.73	26.18 ± 1.11	0.58 ± 0.16	0.27
IsotypeControl-vcMMAE	32.67 ± 19.05	99.00-± 22.18	21.15-± 2.31	0.77-± 0.06	0.33
SS1-vcMMAE	38.50 ± 45.18	109.17 ± 23.08	21.02 ± 1.45	0.57 ± 0.26	0.35
CHS5-vcMMAE	20.67 ± 3.09	104.67 ± 14.21	20.88 ± 1.83	0.57 ± 0.16	0.20
CHS7-vcMMAE	20.67 ± 2.56	99.83 ± 8.55	21.32 ± 1.68	0.79 ± 0.07	0.21
CHS8-vcMMAE	22.83 ± 5.84	105.33 ± 13.61	24.55 ± 1.66	0.48 ± 0.11	0.22
ALA12-vcMMAE	21.17 ± 4.37	94.50 ± 8.52	21.57 ± 1.30	0.58 ± 0.16	0.22

End point represents mean± SD for 6 mice. ALT (alanine aminotransferase), ALP (alkaline phosphatase), BUN (blood urea nitrogen), CRE (creatinine).

Supplementary Table S3. Effects of anti-MSLN IgG1-vcMMAEs on serum biochemical parameters in Capan-2 NOD/SCID mice. Experimental details are shown in Supplementary Methods.

Capan2	*ALT(U/L)*	*ALP(U/L)*	*BUN(mg/dL)*	*CRE(mg/dL)*	*ALT/ALP*
PBS	16.33 ± 1.80	75.83 ± 17.32	25.13 ± 4.32	0.79 ± 0.12	0.22
IsotypeControl-vcMMAE	19.33 ± 8.42	88.17 ± 9.99	20.75 ± 1.30	0.65 ± 0.23	0.22
SS1-vcMMAE	26.67 ± 13.91	102.50 ± 25.17	22.53 ± 11.65	0.82 ± 0.29	0.26
CHS5-vcMMAE	18.83 ± 1.67	98.67 ± 7.87	21.88 ± 0.75	0.77 ± 0.09	0.19
CHS7-vcMMAE	16.67 ± 2.36	107.17 ± 23.13	21.82 ± 2.49	0.64 ± 0.06	0.16
CHS8-vcMMAE	16.00 ± 1.00	87.00 ± 9.18	22.30 ± 1.33	0.64 ± 0.14	0.18
ALA12-vcMMAE	18.50 ± 2.29	92.00 ± 19.51	21.88 ± 2.05	0.62 ± 0.14	0.18

End point represents mean± SD for 6 mice. ALT (alanine aminotransferase), ALP (alkaline phosphatase), BUN (blood urea nitrogen), CRE (creatinine).

IV. References

1 Yu, C. M. *et al.* A panel of anti-influenza virus nucleoprotein antibodies selected from phage-displayed synthetic antibody libraries with rapid diagnostic capability to distinguish diverse influenza virus subtypes. *Scientific reports* **10**, 13318, doi:10.1038/s41598-020-70135-6 (2020).

2 Kuo, W. Y. *et al.* Antibody-drug conjugates with HER2-targeting antibodies from synthetic antibody libraries are highly potent against HER2-positive human gastric tumor in xenograft models. *MAbs* **11**, 153-165, doi:10.1080/19420862.2018.1541370 (2019).

3 Jian, J. W. *et al.* Effective binding to protein antigens by antibodies from antibody libraries designed with enhanced protein recognition propensities. *MAbs* **11**, 373-387, doi:10.1080/19420862.2018.1550320 (2019).

4 Chen, I.-C. *et al.* High throughput discovery of influenza virus neutralizing antibodies from phage-displayed synthetic antibody libraries. *Scientific reports* **7**, 14455, doi:10.1038/s41598-017-14823-w (2017).

5 Hou, S. C. *et al.* High throughput cytotoxicity screening of anti-HER2 immunotoxins conjugated with antibody fragments from phage-displayed synthetic antibody libraries. *Scientific reports* **6**, 31878, doi:10.1038/srep31878 (2016).

6 Tung, C. P. *et al.* Discovering neutralizing antibodies targeting the stem epitope of H1N1 influenza hemagglutinin with synthetic phage-displayed antibody libraries. *Scientific reports* **5**, 15053, doi:10.1038/srep15053 (2015).

7 Chen, H. S. *et al.* Predominant structural configuration of natural antibody repertoires enables potent antibody responses against protein antigens. *Scientific reports* **5**, 12411, doi:10.1038/srep12411 (2015).

8 Yu, C. M. *et al.* Rationalization and design of the complementarity determining region sequences in an antibody-antigen recognition interface. *PLoS One* **7**, e33340, doi:10.1371/journal.pone.0033340 [doi]
PONE-D-11-14795 [pii] (2012).

9 Weitzner, B. D. *et al.* Modeling and docking of antibody structures with Rosetta. *Nat Protoc* **12**, 401-416, doi:10.1038/nprot.2016.180 (2017).

10 Hsu, H.-J. *et al.* Assessing Computational Amino Acid ²-Turn Propensities with a Phage-Displayed Combinatorial Library and Directed Evolution. *Structure (London, England : 1993)* **14**, 1499-1510 (2006).

11 Gorodkin, J., Heyer, L. J., Brunak, S. & Stormo, G. D. Displaying the information contents of structural RNA alignments: the structure logos. *Comput Appl Biosci* **13**, 583-586 (1997).

12 Shoemaker, R. H. The NCI60 human tumour cell line anticancer drug screen. *Nature Reviews Cancer* **6**, 813-823, doi:10.1038/nrc1951 (2006).

13 Fekete, S., Veuthey, J. L., Beck, A. & Guillarme, D. Hydrophobic interaction chromatography for the characterization of monoclonal antibodies and related products. *Journal of pharmaceutical and biomedical analysis* **130**, 3-18, doi:10.1016/j.jpba.2016.04.004 (2016).

14 Stewart, M. J. & Watson, I. D. Standard units for expressing drug concentrations in biological fluids. *British journal of clinical pharmacology* **16**, 3-7 (1983).
